# Selective and Reversible Cation-Gating Adsorption Behavior in Gmelinite Zeolites for Efficient CO_2_ Separation

**DOI:** 10.1021/acsami.5c17473

**Published:** 2025-11-29

**Authors:** Yuto Higuchi, Chihiro Yasuda, Yuna Suetsugu, Satoshi Inagaki, Shunsuke Tanaka

**Affiliations:** † Department of Chemical, Energy and Environmental Engineering, Faculty of Environmental and Urban Engineering, 12860 Kansai University, 3-3-35 Yamate-cho, Suita-shi, Osaka 564-8680, Japan; ‡ Graduate School of Science and Engineering, 12860 Kansai University, 3-3-35 Yamate-cho, Suita-shi, Osaka 564-8680, Japan; § Organization for Research and Development of Innovative Science and Technology (ORDIST), 12860 Kansai University, 3-3-35 Yamate-cho, Suita-shi, Osaka 564-8680, Japan; ∥ Division of Materials Science and Chemical Engineering, 13154 Yokohama National University, 79-5 Tokiwadai, Hodogaya-ku, Yokohama 240-8501, Japan; ⊥ Institute of Advanced Sciences, 13154 Yokohama National University, 79-5, Tokiwadai, Hodogaya-ku, Yokohama 240-8501, Japan; # Carbon Neutrality Research Center (CNRC), 12860 Kansai University, 3-3-35 Yamate-cho, Suita-shi, Osaka 564-8680, Japan

**Keywords:** gmelinite (GME) zeolite, cation-gating, CO_2_ adsorption, ion-exchange, time-resolved PXRD analysis, powder molding

## Abstract

Zeolites exhibiting stepwise adsorption behavior, i.e., a two-step increase in carbon dioxide (CO_2_) uptake, have attracted attention in the field of zeolite research due to the potential to recover CO_2_ using a small amount of energy. In this study, a Na^+^-type gmelinite (GME) zeolite exhibited stepwise adsorption behavior due to the migration of Na^+^ ions in the GME framework. Gas adsorption measurements, in situ powder X-ray diffraction (PXRD) analysis, and magic-angle spinning (MAS) nuclear magnetic resonance (NMR) analysis revealed that Na^+^ serves as a gate-opening cation that induces the migration of CO_2_ from straight channels to the grain-like cages, resulting in notable stepwise adsorption. Remarkably, repetitive CO_2_ adsorption measurements clarified that this stepwise adsorption performance was reversibly induced. Other cation-type GME zeolites such as Li^+^- and K^+^-GME zeolites exhibited type-I CO_2_ adsorption isotherms, indicating only Na^+^ ions were capable of inducing the cation-gating CO_2_ adsorption performance. Furthermore, time-resolved PXRD analysis revealed that the migration rate of Na^+^ was high under the CO_2_ adsorption and desorption processes. In addition, stepwise adsorption behavior was observed even when the GME zeolite powder was pelletized. These findings pave the way for the development of highly efficient CO_2_ separation processes that rely on zeolites with stepwise adsorption properties.

## Graphical abstract

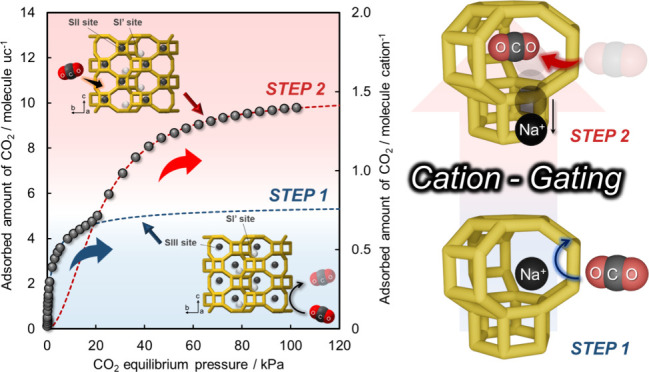

## 1 Introduction

Carbon dioxide (CO_2_) is a greenhouse gas that is driving climate change worldwide. For example, flue gases with high CO_2_ concentrations are emitted from power plants and chemical facilities in industry. Consequently, the development of effective CO_2_ separation processes has received considerable interest among researchers aiming to address this pressing global issue.

With respect to the materials used in CO_2_ separation processes, zeolites, which constitute a class of microporous materials, possess ordered nanostructures and homogeneous pore diameters, which endow them with the ability to selectively adsorb CO_2_ molecules from mixed gases containing N_2_ and CH_4_ molecules.^1–9^ Compared with other porous materials, zeolites have been demonstrated to exhibit superior CO_2_ adsorption capacity because of the electrostatic interaction between CO_2_ molecules and extra-framework cations, which generates an electric field within the zeolite structure. Therefore, the CO_2_ uptake by zeolites (e.g., zeolite 13X) increases sharply at low pressures. However, a substantial amount of energy is required for CO_2_ recovery via the pressure swing adsorption (PSA) process because of the broad pressure swing operation range between high-pressure and vacuum conditions.^10–13^ Consequently, the establishment of an energy-saving CO_2_ recovery process using zeolites or other CO_2_ adsorbents in which large amounts of CO_2_ molecules can be desorbed within a narrow pressure range is important for researchers.

Flexible metal–organic frameworks (flexible MOFs), which constitute a class of microporous materials comprising metal ions/clusters and organic compounds that exhibit stepwise adsorption due to structural transitions, have been reported.^14–26^ The phenomenon of stepwise adsorption involves a sharp increase in gas uptake at a specific pressure, termed gate-opening adsorption. Owing to the structural specificity of flexible MOFs, such adsorption can be categorized into various forms, including “linker rotation,” ^14,15^ “breathing,”^16^ and “stacking layer expansion”.^17–19^ These unique adsorptions are generally triggered by structural transitions of the MOF structures.

In contrast, in the field of zeolite research, stepwise adsorption due to the migration of extra-framework cations in zeolite micropores has been reported.^27–44^ These extra-framework cations that impede the diffusion of guest molecules through the pores are termed “door-opening/closing cations” and are located at sites where they block guest molecules from entering zeolite pores. Therefore, in general, guest molecules cannot be adsorbed in zeolites because of the obstruction of pores by door-opening/closing cations. However, these cations can migrate from one cation site to another without affecting the diffusion path of the guest molecules. Such migration occurs in conjunction with the desorption of molecules such as H_2_O previously adsorbed in micropores via an activation process. Guest molecules were then adsorbed in zeolite pores. This adsorption phenomenon has been referred to as the “trapdoor” phenomenon and has been observed in Cs^+^-chabazite (CHA)- and Cs^+^-RHO-type zeolites in previous studies.^27–35^ In the adsorption process, small pores such as eight-ring windows (8RWs), large spaces that are accessible through 8RWs, such as CHA and Linde type-A (LTA) cages, and the positioning of large cations such as Cs^+^ ions in the cages are crucial for trapdoor adsorption. Additionally, gismondine (GIS)-, merlinoite (MER)-, and phillipsite (PHI)-type zeolites exhibit a cation-breathing adsorption mechanism, whereby framework transition and the migration of extra-framework cations in the pores occur simultaneously during CO_2_ adsorption/desorption.^36–42^ In this phenomenon, the greater the amount of Al in the zeolite framework, the more flexible the zeolite framework becomes in the adsorption process. These phenomena occur in zeolites with 8RWs that contain large cations such as Rb^+^ and Cs^+^ ions. To elucidate the unique adsorption mechanisms in terms of the relationship between CO_2_ adsorption and the zeolite structure, in situ powder X-ray diffraction (PXRD) analysis under a CO_2_ atmosphere is necessary. Another report described the effects of the trapdoor framework (CHA and MER) and door-opening/closing cations on the gas adsorption performance using in situ PXRD analysis.^43^

In this report, gmelinite (GME) zeolite was synthesized from a H^+^-type faujasite (FAU) zeolite as an aluminosilicate source. Previously, the synthesis,^45–51^ aluminum distribution,^52^ adsorption,^45,46,48^ and ion-exchange^53^ properties of GME zeolites have been reported. In addition, a recent report revealed that Na^+^-type GME zeolites with Si/Al ratios ranging from 20 to 40 showed high CO_2_ adsorption capacities.^45^ However, these Na^+^-GME zeolites exhibited type-I CO_2_ adsorption isotherms, which are typical of zeolites that do not exhibit dramatic structural changes upon CO_2_ adsorption. In contrast, the Na^+^-GME zeolite with a Si/Al ratio of 2.42, synthesized in this study, exhibited unprecedented stepwise adsorption, in which the CO_2_ uptake increased in two steps. Notably, only Na^+^-type GME zeolite exhibited stepwise CO_2_ adsorption behavior; CO_2_ uptake first increased sharply at low CO_2_ pressures and then increased further beyond the threshold pressure. To clarify the stepwise adsorption mechanism, gas (CO_2_, N_2_, and CH_4_) and vapor (H_2_O) adsorption measurements and in situ PXRD analysis under a CO_2_ atmosphere were conducted for Li^+^, Na^+^, and K^+^-GME zeolites to investigate the effect of the class of cations on the adsorption performance. In addition, ^23^Na magic-angle spinning (MAS) nuclear magnetic resonance (NMR) analysis was performed to estimate the locations of Na^+^ within the GME framework. According to the analysis, this intriguing stepwise adsorption isotherm, which can be attributed to the presence of Na^+^ ions, indicates cation-gating adsorption. In addition, compared with CHA, MER, RHO, and PHI zeolites, the Na^+^-GME zeolite showed high CO_2_ adsorption capacity at *P*_CO2_ = 101.3 kPa. The impact of pelletization on the CO_2_ adsorption performance was also evaluated in this study, revealing that the stepwise adsorption behavior is maintained in molded zeolite forms, which is a previously unreported finding. The cation-gating stepwise adsorption mechanism and the effect of pelletization on adsorption were investigated in detail, thereby revealing new possibilities for highly efficient CO_2_ recovery processes using GME zeolites.

## 2 Experimental Section

### 2.1 Characteristics of GME Zeolites

The crystal structures of the products were determined through PXRD analysis on a MiniFlex 600 instrument (Rigaku) with CuKα radiation (wavelength: λ = 1.5406 Å). PXRD data were collected from 3° to 60° with a scan speed of 5.0°/min and a step size of 0.02 degrees. The particle morphology of the GME zeolite was observed through field emission-scanning electron microscopy (FE-SEM) on an S-4800 instrument (Hitachi High-Tech). The accelerating voltage was 2.0 kV, and the working distance was set to 9.7 mm. ^13^C DDMAS, ^23^Na MAS, ^27^Al MAS, and ^29^Si DDMAS NMR spectra were recorded with resonance frequencies of 12, 10, 12, and 8 kHz (AV4400; Bruker). The relaxation time for ^13^C DDMAS, ^23^Na MAS, and ^29^Si DDMAS was 64 s, and that for ^27^Al MAS was 1 s. The accumulation time for ^27^Al MAS was 64 s, while that for the other methods was 1024 s. The reference material for ^13^C DDMAS, ^29^Si DDMAS was tetramethylsilane, while aluminum chloride and sodium chloride were used for ^27^Al MAS and ^23^Na MAS, respectively. The ^29^Si DDMAS NMR spectrum was separated into various peaks, and these peak areas were calculated to obtain the Si/Al ratio of the GME zeolites via eq 1:

$$(\mathrm{Si}/\mathrm{Al}{)}_{\mathrm{NMR}}=\sum_{n=0}^{4}I_{n}/\sum_{n=0}^{4}(n/4)I_{n}$$ 1

where *I_n_* is the intensity of the Q^4^ (nAl) spectrum. The Na^+^- and Na^+^/K^+^-GME zeolites were activated using BELPREP VAC II and III (MicrotracBel Corp., Japan) at 498 K for 4 h under pressures lower than 10 Pa in the sample cells. Afterward, the sample was exposed to Ar, N_2_, and CO_2_ atmospheres at 298 K and a total pressure of 100 kPa in a glovebox containing desiccants (the sample was exposed to a CO_2_ atmosphere for 10–30 min to reach adsorption equilibrium). The CO_2_ partial pressure was controlled in the range of 5–50 kPa, with the balance made up by Ar to maintain a total pressure of 100 kPa. The sample was subsequently inserted into the measurement cell for NMR analysis. The cation ratios of the as-prepared and ion-exchanged GME zeolites were determined via inductively coupled plasma spectroscopy (ICP-AES) (Shimadzu) and EDX on an Emax EVOlution instrument (Horiba) attached to an S-4800 instrument (Hitachi High-Tech). The SEM emission voltage for EDX analysis was 15 kV, and the working distance was 15 mm. CO_2_, N_2_, and CH_4_ adsorption measurements at 298 and 318 K were performed on a BELSORP-max instrument (MicrotracBel Corp., Japan). The GME zeolites were degassed using BELPREP VAC II and III (MicrotracBel Corp., Japan) at 498 K for 4 h under a pressure lower than 10 Pa in the sample cells. Equilibrium was attained when the gas pressure in the sample cell changed to less than 0.3% over 180 s. Analysis methods for CO_2_ adsorption isotherms were described in the Supporting Information. CO_2_-TPD measurements were performed for the Li^+^-GME and Na^+^-GME zeolites on a BELCAT II instrument (MicrotracBel Corp., Japan). A 0.1 g sample was placed in a glass column (internal diameter: 8 mm), and a thermocouple was inserted into the glass tube (external diameter: 3 mm). The samples were degassed at 498 K for 4 h at a He flow rate of 20 mL/min (STP), and 2% CO_2_/He and 100% CO_2_ gas at 101.3 kPa were then continuously flowed at a rate of 20 mL/min (STP) into the samples in the glass column. After the CO_2_ adsorption equilibrium was attained, He gas was flowed into the sample at 20 mL/min (STP), and the temperature was maintained at 298 K for 5–6 h. Afterward, the temperature was increased from 298 to 773 K at a heating rate of 2 K/min, with He gas flowing at the same rate. The CO_2_ concentration at the outlet was measured via a thermal conductivity detector (TCD) on a BELCAT II instrument.

### 2.2 In Situ PXRD Analysis under a CO_2_ Atmosphere

The crystal structures of the GME zeolites during CO_2_ adsorption and desorption were recorded using in situ high-resolution PXRD (HR-PXRD) equipment with λ = 0.0799 nm on beamline 02B2 (BL02B2 at the SPring-8 facility, Japan). The samples were placed in a 0.5 mm diameter borosilicate glass capillary, and the capillary was then attached to the equipment. The temperature was controlled by using low-temperature N_2_ spraying equipment. The CO_2_ pressure was controlled by a remote gas pressure controller. All of the samples were degassed using a BELPREP VAC III (MicrotracBel Corp., Japan) at 498 K under a pressure lower than 10 Pa for 4 h before CO_2_ gas was introduced into the capillary. Equilibrium was assessed at each CO_2_ pressure on the basis of the absence of a change in the diffraction pattern for 1 h.

### 2.3 TR-PXRD Measurements

Time-resolved PXRD (TR-PXRD) analysis of the Na^+^-GME zeolite was performed using HR-PXRD equipment with λ = 0.0799 nm installed on BL02B2 at the SPring-8 facility, Japan. The sample was degassed at 498 K for 4 h before PXRD analysis. The sample was placed in a 0.5 mm diameter borosilicate glass capillary and attached to in situ HR-PXRD equipment. The capillary was attached to a remote gas handling system, and CO_2_ gas was introduced at a pressure of approximately 50 kPa into the sample in the capillary 20 s after the start of X-ray exposure. PXRD patterns were recorded every 1 s throughout the X-ray exposure process. The adsorption temperature was maintained at 298 K by using a N_2_ gas blower. After the measurement under CO_2_ adsorption, the capillary containing the sample was evacuated 20 s after the start of the X-ray exposure. PXRD patterns were recorded every 1 s throughout the X-ray exposure process.

## 3 Results and Discussion

### 3.1 Physicochemical Characteristics of GME Zeolites

First, Na^+^-GME zeolite was synthesized via a steam-assisted interzeolite transformation process using an H^+^-type FAU zeolite, based on the synthesis method proposed by Mielby et al.^49^ Ion exchange of the as-synthesized GME zeolite from Na^+^ to Li^+^ and K^+^ ions was subsequently performed. PXRD analysis (Figure 1a) revealed that the GME framework remained unchanged during the ion exchange treatment, irrespective of the extra-framework cations in the pores. The intensity of the PXRD peaks of K^+^-GME exhibited a slight change in comparison with that of Na^+^-GME zeolite, attributable to the high scattering of X-rays induced by the introduction of large cations such as K^+^ ions.^54,55^ On the other hand, the PXRD patterns of Na^+^-GME zeolite obtained by ion-exchange from K^+^-GME zeolite showed the same PXRD pattern as that of Na^+^-GME zeolite, indicating that the structure of GME zeolite was preserved before and after the ion-exchange process (Figure S1). The pore characteristics of the as-synthesized and ion-exchanged GME zeolites were determined via N_2_ adsorption and desorption isotherms obtained at 77 K (Figure 1b). The data are listed in Table 1. The chemical formula was obtained using the Si/Al ratios determined from the ^29^Si dipolar decoupling magic-angle spinning (DDMAS) NMR spectrum (Figure S2a), and the cation ratios were determined through inductively coupled plasma atomic emission spectrometry (ICP-AES) and energy-dispersive X-ray spectrometry (EDX) analysis. All the Al atoms in the GME framework were tetra-coordinated (δ_iso_ = 60.0), and no hexa-coordinated Al atoms were detected (Figure S2b). The Brunauer–Emmett–Teller surface area (*S*_BET_) and micropore volume (*V*_micro_) of the Na^+^- and K^+^-GME zeolites, calculated from the N_2_ adsorption–desorption isotherms based on the Rouquerol criteria, were notably lower than those of the Li^+^-GME zeolite. These results indicate that Na^+^ and K^+^ ions are located in the straight channel and at the pore entrance of the gme cage, thereby impeding the diffusion of N_2_ molecules into the micropores. In contrast, N_2_ molecules were adsorbed in the Li^+^-GME zeolite, resulting in a *V*_micro_ value of 0.32 cm^3^ g^–1^. Here, the ideal pore volumes of the straight channel and the gme cage of the Li^+^-GME zeolite were calculated to confirm the N_2_ adsorption sites within the framework. The calculations indicate that the pore volumes of the straight channel and the gme cage of the Li^+^-GME zeolite were 0.15 and 0.29 cm^3^ g^–1^, respectively. These calculations are based on the calculation results of the volume of the void space of the GME framework, which is subtracted from the volume of Li^+^ ions (ionic radius: 0.60 Å^56^) (Figure S3). The arrangement of Li^+^ ions in the GME zeolite is discussed in the following section. The calculation results suggest that N_2_ molecules can be adsorbed in the straight channels and gme cages in the Li^+^-GME zeolite. Afterward, the cation sites in the GME framework were considered on the basis of the N_2_ adsorption measurement results. The GME framework comprises double 6-rings (d6rs) and possesses a 12-ring window with a size of 7.0 × 7.0 Å connected to a straight channel and an 8-RW with a size of 3.6 × 3.9 Å that connects the straight channel and the gmecage^57^ (Figure 1c). In addition, the as-prepared Na^+^-GME zeolite exhibits a hexagonal prism morphology (Figure S4), which is consistent with the GME framework topology.

**Figure 1:**
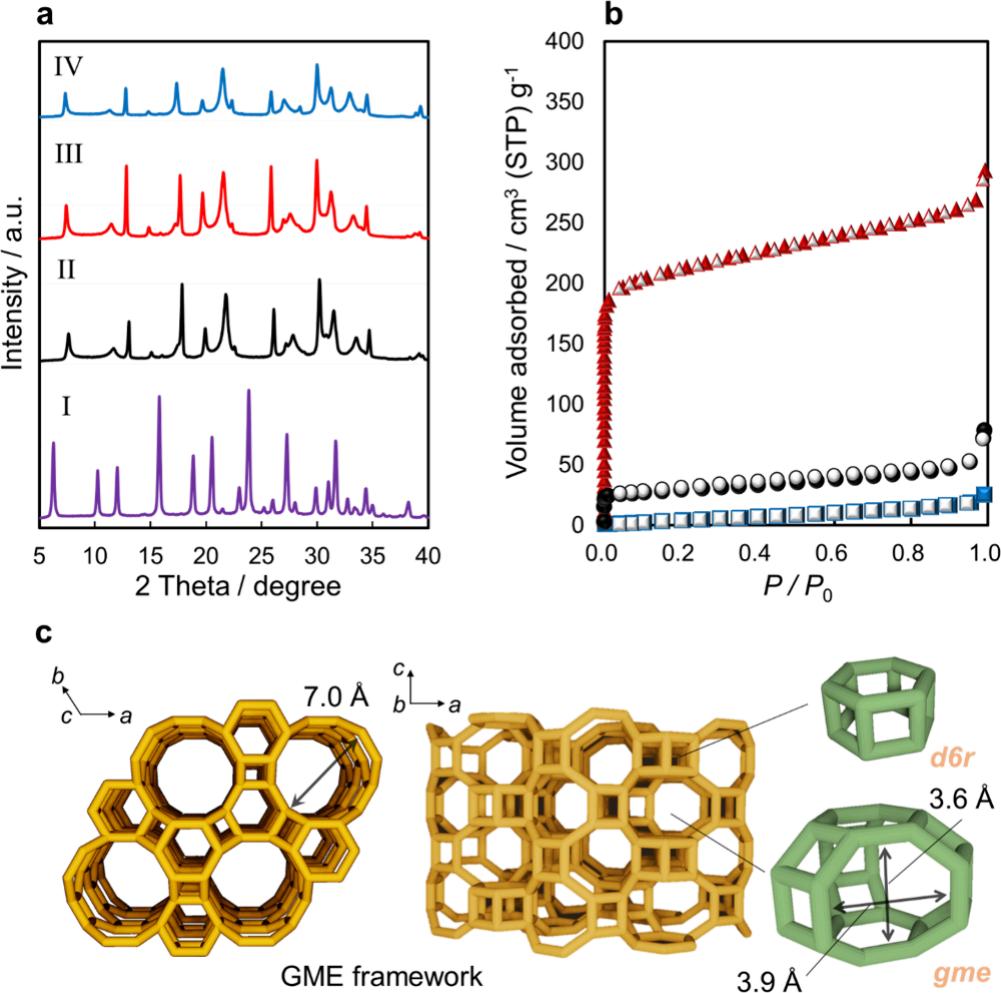
Crystal structure and N_2_ adsorption performance at 77 K of the as-prepared and ion-exchanged GME zeolites. (a) PXRD patterns of the (I) H^+^-FAU, (II) Na^+^-, (III) Li^+^-, and (IV) K^+^-GME zeolites. (b) Nitrogen adsorption and desorption isotherms of the (▲) Li^+^-, (●) Na^+^-, and (■) K^+^-GME zeolites at 77 K (closed symbol: adsorption; open symbol: desorption). (c) Framework of the GME-type zeolite.^57^

**Table 1:** Chemical Composition and Pore Characteristics of GME Zeolites^a^

sample	chemical formula^b^	*S*_BET_ /m^2^ g^–1^ ^c^	*V*_micro_/cm^3^ g^–1^ ^d^
Li^+^-GME	Li_6_Na[Al_7_Si_17_O_48_]	807	0.32
Na^+^-GME	Na_7_[Al_7_Si_17_O_48_]	66	0.025
K^+^-GME	K_7_[Al_7_Si_17_O_48_]	2.9	0.0022

a The Si/Al ratio calculated on the basis of ^29^Si DDMAS NMR spectra is 2.42.

b Chemical composition determined via ICP analysis and ^29^Si DDMAS NMR measurements.

c *S*_BET_ calculated via the Brunauer–Emmett–Teller (BET) method using N_2_ adsorption isotherms at 77 K.

d *V*_micro_ calculated via the *t*-plot method using N_2_ adsorption isotherms at 77 K.

### 3.2 CO_2_ Adsorption Performance and Locations of Cations in GME Zeolites

CO_2_ adsorption and desorption measurements at 298 K were conducted to investigate the performance of the CO_2_ adsorption of the GME zeolites (Figures 2a–c and S5). The Li^+^- and K^+^-GME zeolites exhibited type-I adsorption isotherms. The CO_2_ uptake first increased at low pressures and then reached saturation. In contrast, the Na^+^-GME zeolite exhibited a stepwise CO_2_ adsorption isotherm, with a steep increase in CO_2_ uptake at low pressures and a subsequent stepwise increase at *P*_CO2_ = 18 kPa. The adsorption capacity of the Na^+^-GME zeolite was notably greater than that of the Na^+^-FAU zeolite, which comprises d6r units with Si/Al = 2.69. Moreover, the adsorption behavior of the Na^+^-GME zeolite differed from that of the Na^+^-FAU zeolite precursor (Figure S6 and Table S1). It should be noted that the CO_2_ uptake of Na^+^-GME zeolite exhibits variation depending on the samples, ranging from 5.5 to 6.4 mmol g^–1^ due to the influence of crystallinity and the porosity of the H^+^-FAU zeolite used as the starting material. In addition, the result of reversible ion exchange from K^+^-GME to Na^+^-GME zeolites was equivalent to that of the as-prepared Na^+^-GME zeolite in terms of CO_2_ adsorption and desorption (Figure 2d). These findings indicate that the cation type in the GME zeolite influences the CO_2_ adsorption performance, with Na^+^ ions playing a pivotal role in shaping CO_2_ stepwise adsorption in the GME zeolite. Interestingly, the CO_2_ adsorption performance of the Na^+^-GME zeolite was reversible during the CO_2_ adsorption and desorption (Figure S7). As shown in Figure 2d, Na^+^ ions were found to be crucial for the stepwise CO_2_ adsorption behavior exhibited by the GME zeolite. The uptakes of N_2_ and CH_4_ were lower than that of CO_2_ in each GME zeolite (Figure S8). Furthermore, N_2_ and CH_4_ molecules were not adsorbed in a stepwise manner in the GME zeolites. These findings suggest that Na^+^ ions and CO_2_ guest molecules triggered stepwise adsorption in the GME zeolites. The observed CO_2_ and N_2_ adsorption/desorption behavior suggests that Na^+^ ions were primarily located at the SII or SIII sites (Figure S9), thereby impeding the entry of N_2_ molecules into the straight channels. The following section will address details of the cation sites. K^+^ ions were also located at SIII sites, considering the CO_2_ diffusion path in straight channels, and K^+^ ions completely blocked the 8RWs of the gme cages. In contrast, in the case of Li^+^-GME zeolite, N_2_ molecules could be adsorbed in the straight channels and gme cages, suggesting that Li^+^ ions were located at SII sites.

**Figure 2:**
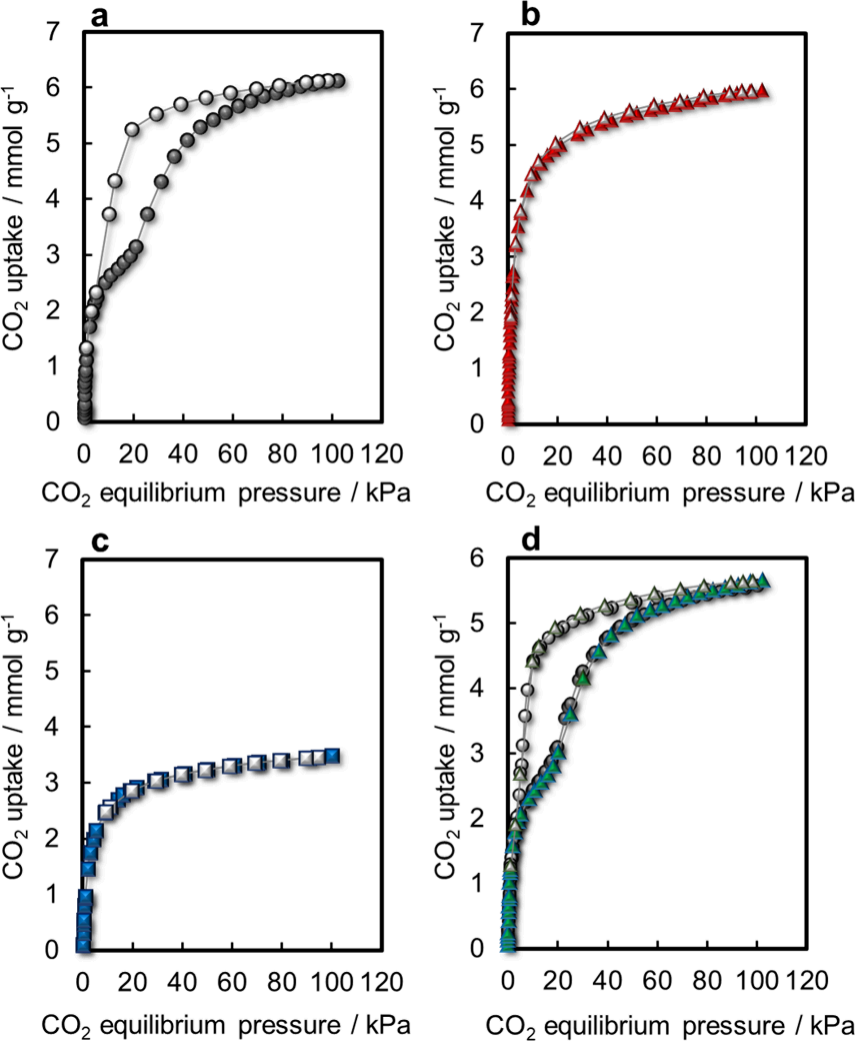
CO_2_ adsorption and desorption isotherms at 298 K. (a) As-prepared Na^+^-GME, (b) Li^+^-GME, and (c) K^+^-GME zeolites. (d) (●) As-prepared Na^+^-GME and (▲) ion-exchanged Na^+^-GME zeolites from K^+^-GME zeolite (closed symbol: adsorption; open symbol: desorption).

### 3.3 Identification of the Location of Na^+^ Ions in the GME Framework

The locations of Na^+^, Li^+^, and K^+^ ions in the GME framework were investigated to clarify the stepwise CO_2_ adsorption mechanism. The GME framework contains only one tetrahedral site, namely, T1, indicating that the cation sites within this framework can be estimated. The GME framework is proposed to include five types of cation sites: SI, SI′, SII, SII′, and SIII (see Figures 3a and S10). These designations are based on the topology of the GME framework and the crystal structure reported in previous studies.^58^ The SI, SI′, SII, SII′, and SIII sites are located on the d6r, at the center of the d6r window, beside the 4-ring window (4RW) on the side of the straight channel, beside the 4RW on the side of the gme cage, and at the center of the 8RW, respectively. In addition, guest molecules could mainly diffuse into 12-ring straight channels from the external surface of zeolite particles. Therefore, the N_2_ adsorption/desorption behavior at 77 K suggested that Na^+^, Li^+^, and K^+^ were located at SII or SIII sites. ^23^Na MAS NMR analysis of the Na^+^-GME zeolite was performed to estimate the distribution of the cation sites within the GME framework. Before the desorption of H_2_O through the activation process, each ^23^Na MAS NMR spectrum (Figure 3b) revealed the presence of Na^+^ ions interacting with H_2_O molecules, suggesting that the environment around the Na^+^ ions was equivalent to that around the Na^+^ ions located at different sites (Figure 3b-I). The spectrum of the Na^+^-GME zeolite activated in an Ar atmosphere (Figure 3b-II) was resolved into three peaks at δ_iso_ = 12.2, 4.1, and −20.0 ppm, indicating that Na^+^ cations were located at three distinct cation sites in the GME framework after the removal of H_2_O molecules. GME zeolites with various Na^+^/K^+^ ratios were prepared to determine the location of the Na^+^ ions assigned to each peak (Figure S11). The peak at −19.2 ppm was weakened with decreasing Na^+^/Al molar ratio, in accordance with the decrease in the amount adsorbed in the second step (Figure S12). Compared with those at the other cation sites, Na^+^ at the SIII site could be briefly ion-exchanged. Therefore, the peak at −19.2 ppm was assigned to Na^+^ at the SIII site, contributing to the CO_2_ adsorption behavior in the second step. In addition, the peak area at δ_iso_ = 12.2 ppm decreased along with that at δ_iso_ = −20.0 ppm, suggesting that the signal at δ_iso_ = 12.2 ppm could be assigned to the SII site, which is located in the straight channel. Additionally, the total peak area ratio of the Na^+^-GME zeolite between δ_iso_ = −20.0 and 12.2 ppm reached 82.0%, which suitably agrees with the proportion of Na^+^ ions at the SII and SIII site in a GME unit cell (85.7%), as detailed in Table 1 and shown in Figure S9. The proportion of the peak area for δ_iso_ = 4.1, which can be attributed to Na^+^ at the SI’ site in the gme cage, increased. In contrast, the presence of more than 50% of K^+^ in the GME zeolite complicated the environment of the Na^+^ ions because those samples exhibited only one signal in their ^23^Na NMR spectra.

**Figure 3:**
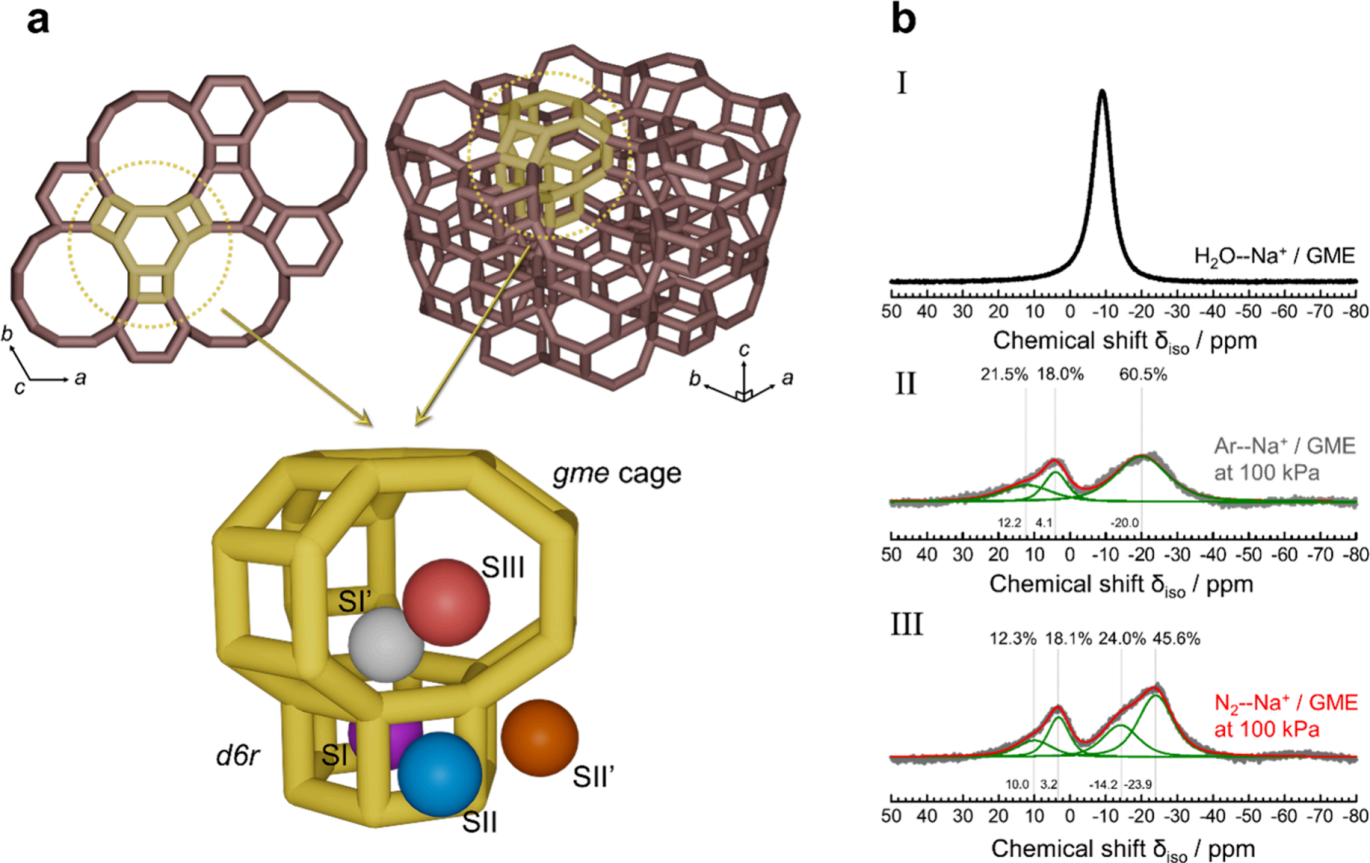
Locations and states of Na^+^ in GME zeolite. (a) Cation sites in the GME framework. (b) ^23^Na MAS NMR spectra of Na^+^-GME; (I) hydrated, (II) dehydrated under an Ar atmosphere, and (III) dehydrated under a N_2_ atmosphere.

Next, the spectrum of Na^+^-GME zeolite pretreated in a N_2_ atmosphere (Figure 3b-III) was resolved into four peaks, i.e., δ_iso_ = 10.0, 3.2, −14.2, and −23.9 ppm, which differ from those resolved under an Ar atmosphere because of the interaction between Na^+^ and N_2_ with a quadrupole moment.^59^ The peak at δ_iso_ = −20.0 ppm in the Na^+^-GME zeolite exposed to N_2_ was resolved into two peaks at δ_iso_ = −14.2 and −23.9 ppm. There were two distinct environments around Na^+^ at the SIII site. Considering the N_2_ adsorption isotherm of the Na^+^-GME zeolite at 298 K (Figure S8a), the peaks at δ_iso_ = −14.2 and −23.9 ppm indicated that Na^+^ interacted with N_2_ and was present alone at the SIII site (Figure S13). In addition, the adsorbed amount of N_2_ per unit cell and per cation was determined to be 0.6 and 0.1, respectively, suggesting that a small amount of N_2_ interacted with Na^+^ ions. Na^+^-GME under a CO_2_ atmosphere was also analyzed via both ^23^Na MAS and ^13^C DDMAS NMR analyses under different partial CO_2_ pressures balanced with Ar (Figure S14). The ^23^Na NMR peaks under an Ar atmosphere at 100 kPa (Figure 3b-II) were analogous to those under a 5% CO_2_/Ar atmosphere at 100 kPa (Figure S14a), but the peak assigned to the SIII site changed slightly with CO_2_ adsorption, suggesting that CO_2_ interacted with Na^+^ at the SIII site at low CO_2_ partial pressures. Additionally, a single NMR peak was observed at δ_iso_ = −21.7 ppm under CO_2_ pressure at 100 kPa (Figure S14b), indicating that all of the Na^+^ ions in the GME framework interacted with CO_2_. In contrast, ^13^C DDMAS NMR analysis revealed that the peak position of the NMR peak was maintained under CO_2_ pressure (Figure S14c,d). In other words, the adsorption state of CO_2_ on Na^+^ in the GME framework was independent of the amount of CO_2_ adsorbed.

### 3.4 GME Crystalline Structures under a CO_2_ Atmosphere

In situ PXRD analysis was applied to the as-prepared and ion-exchanged GME zeolites under CO_2_ adsorption to investigate the effects of CO_2_ adsorption and desorption on the change in the GME crystalline structure (Figures 4, S15, and S16). The crystal structure of the Na^+^-GME zeolite notably changed throughout the dehydration and CO_2_ adsorption/desorption processes. Furthermore, the PXRD patterns obtained under CO_2_ adsorption in step 2 (nos. 3 and 4) conformed with those obtained under H_2_O saturation (hydrated Na^+^-GME zeolite). Finally, the PXRD pattern of the Na^+^-GME zeolite after CO_2_ desorption reverted to the original dehydrated form (no. 1). In contrast, the PXRD patterns of the Li^+^-GME and K^+^-GME zeolites (Figures S15 and S16, respectively) remained unchanged in the same process. Therefore, guest molecules such as H_2_O and CO_2_ affect only the structure of the Na^+^-GME zeolite. This distinct change in the PXRD pattern observed in the Na^+^-GME zeolite represents an intriguing characteristic. Notably, the results suggest that the Na^+^ ions in the GME framework migrate among the cation sites during the CO_2_ adsorption and desorption process because the Li^+^-GME and K^+^-GME zeolites did not undergo a framework transition in the same process. Time-resolved PXRD (TR-PXRD) analysis of the Na^+^-GME zeolite (Figure 5a–c) was performed to confirm the cation-gating rate during the CO_2_ adsorption and desorption process, revealing that the PXRD patterns changed rapidly upon the introduction of CO_2_. The change in the PXRD pattern for the structure of the Na^+^-GME zeolite was completed after 160 s (Figure 5a). Conversely, upon a reduction in the CO_2_ pressure to a vacuum, the PXRD pattern (Figure 5b) began to change rapidly, completely reverting to the original pattern within 40 s from the onset of evacuation. These TR-PXRD changes agreed with the results of in situ PXRD analysis under CO_2_ adsorption/desorption for the Na^+^-GME zeolite (Figure 4a).

**Figure 4:**
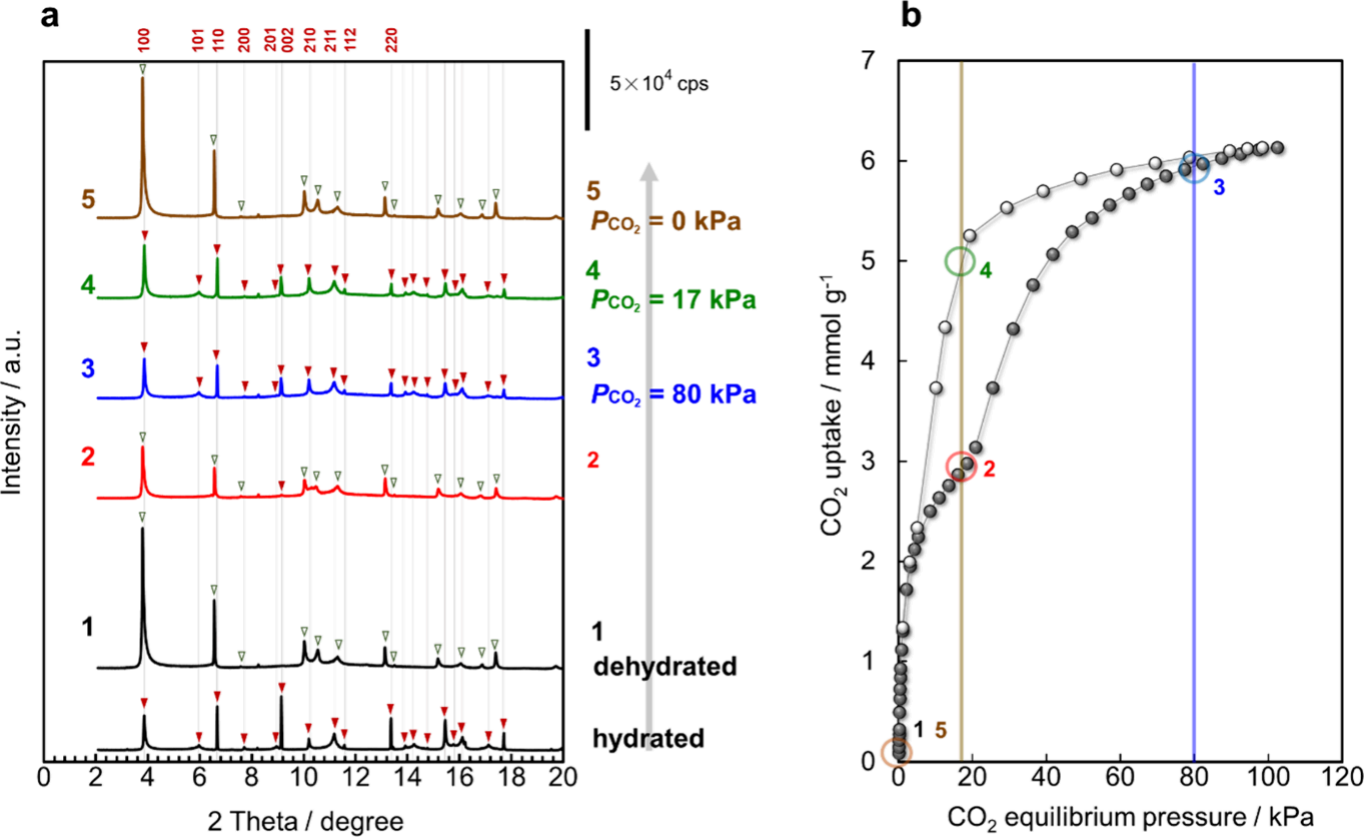
In situ PXRD analysis of the Na^+^-GME zeolite in static and dynamic CO_2_ adsorption processes. (a) In situ PXRD patterns during dehydration and CO_2_ adsorption/desorption at 298 K (λ = 0.0799 nm). (▼) GME framework incorporating CO_2_ or H_2_O molecules. (▽) GME framework after the desorption of guest molecules. (b) Points on the CO_2_ adsorption/desorption isotherms corresponding to the introduction of CO_2_ pressure during the in situ PXRD measurements.

**Figure 5:**
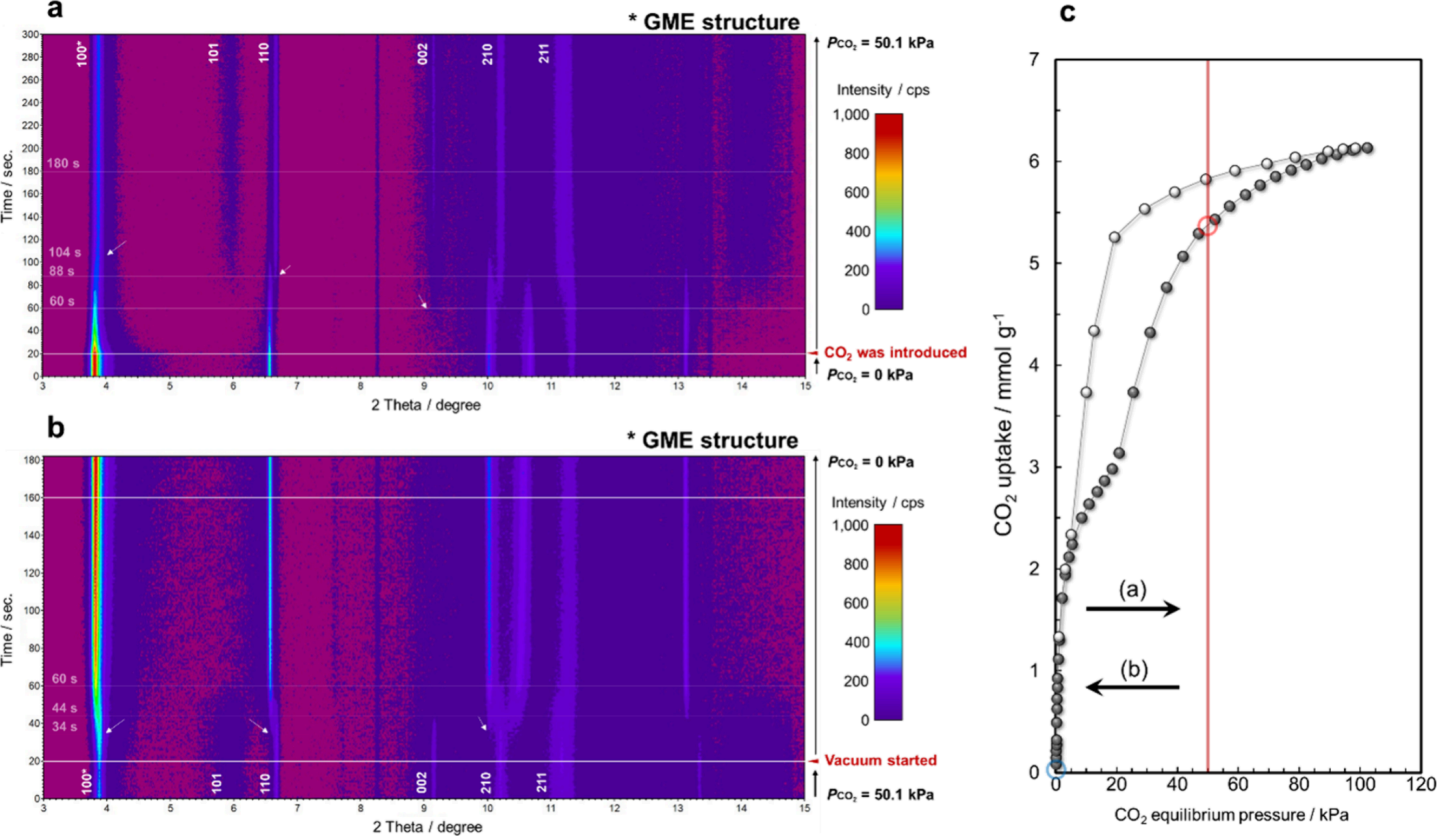
Time-resolved in situ PXRD patterns during CO_2_ adsorption and desorption at 298 K (λ = 0.0799 nm, scan rate of 1 s^–1^). (a) CO_2_ adsorption process (vacuum → CO_2_ pressure of 50.1 kPa). (b) CO_2_ desorption process (CO_2_ pressure of 50.1 → 0 kPa). (c) Points on the CO_2_ adsorption/desorption isotherms corresponding to the introduction of CO_2_ pressure during time-resolved in situ PXRD measurements.

### 3.5 Calculation of the Amount of CO_2_ Adsorbed in GME Zeolites

The CO_2_ adsorption isotherm of the Na^+^-GME zeolite was analyzed via the Langmuir–Freundlich (L–F) equation (eq 2), and the CO_2_ saturation capacity and amount of CO_2_ adsorbed per cation and unit cell were calculated to estimate the number of CO_2_ adsorption sites in the GME zeolite (Figure S17a and Table 2). The CO_2_ adsorption isotherms were divided into 2 steps: The first step was characterized by an initial increase in the level of CO_2_ uptake prior to *P*_CO2_ = 18 kPa, and the second step corresponded to a subsequent increase in the level of CO_2_ uptake after *P*_CO2_ = 18 kPa. As detailed in Table 2, the CO_2_ saturation capacities in steps 1 and 2 were 3.5 and 2.9 mmol g^–1^, respectively. The total amount of CO_2_ adsorbed in these steps was 6.4 mmol g^–1^. The amounts of CO_2_ adsorbed per cation in steps 1 and 2 were 0.8 and 1.5 molecules cation^–1^, respectively. Here, according to the Si/Al ratio and cation positions within the GME framework, CO_2_ molecules could not diffuse to the gme cage because of the presence of Na^+^ ions at the SIII sites in the first step. In other words, CO_2_ molecules could not interact with Na^+^ ions in the glycerol cage in the first step. ^23^Na MAS NMR spectra of the Na^+^-GME zeolite under a CO_2_ atmosphere, wherein the CO_2_ partial pressure was controlled with Ar, suggested that CO_2_ interacted with only Na^+^ ions at the SIII site in the first step (Figure S18). The signal at δ_iso_ = −20.0 (SIII site) changed drastically in the CO_2_ pressure range from 5 to 15 kPa compared with the signals at δ_iso_ = 4.1, attributed to Na^+^ ions at the SI’ site, indicating that CO_2_ initially interacted with Na^+^ ions at the SIII site. In addition, the results indicated that the Na^+^ ions present at the SI site exhibited no reactivity regarding the CO_2_ interaction. Furthermore, all the signals changed with the increase in CO_2_ pressure above 30 kPa, corresponding to the second step of the CO_2_ adsorption isotherm of Na^+^-GME zeolite, and finally, a single signal was observed, suggesting that all Na^+^ ions in GME zeolite interacted with CO_2_ molecules in the second step. On the basis of these results, the practical number of CO_2_ molecules adsorbed in the GME zeolite in step 1 was determined as 0.9 molecules cation^–1^. The number of adsorbed CO_2_ molecules per unit cell in step 1 was determined as 5.6 molecules uc^–1^, indicating that one CO_2_ molecule was adsorbed onto one Na^+^ in the GME zeolite. In contrast, the total number of CO_2_ molecules adsorbed across all steps was 10.2 molecules uc^–1^. Furthermore, the CO_2_ saturation capacity and the number of adsorbed CO_2_ molecules per cation and unit cell in the Li^+^- and K^+^-GME zeolites were calculated (Figure S17b and Table 3). In the case of the Li^+^-GME zeolite, the numbers of CO_2_ molecules per unit cell and cation were 9.4 and 1.3, respectively, which are consistent with those of the Na^+^-GME zeolite across all of the steps. Li^+^ ions were located at the SII sites and did not block the entry of CO_2_ molecules into the gme cages. Therefore, CO_2_ molecules were adsorbed in the straight channels and gme cages in the Li^+^-GME zeolite throughout the entire process. In the case of the K^+^-GME zeolite, the number of CO_2_ molecules per cation was 1.0, which is consistent with that of the Na^+^-GME zeolite in step 1. K^+^ ions were located at the SIII sites, which are the same positions as those for the Na^+^ ions, indicating that CO_2_ molecules were adsorbed exclusively in the straight channels for both the Na^+^-GME zeolite in step 1 and the K^+^-GME zeolite.

**Table 2:** Adsorbed Amount of CO_2_ Per Gram, Unit Cell, and Cation of the Na^+^-Type GME Zeolite^a^

step	*Q* (CO_2_)_sat._/mmol g^–1^	*Q* (CO_2_)/molecules uc^–1^	*Q* (CO_2_)/molecules cation^–1^
1	3.5	5.6	0.9^b^
2	2.9	4.6	
1 + 2	6.4	10.2	1.5

a Adsorbed amount of CO_2_ obtained via the analysis of CO_2_ adsorption isotherms using the Langmuir–Freundlich equation.

b The number of CO_2_ molecules per cation was calculated using six cations in the straight channel because CO_2_ molecules could not be adsorbed onto cations in the gme cage.

**Table 3:** Adsorbed Amount of CO_2_ Per Gram, Unit Cell, and Cation of the Li^+^- and K^+^-Type GME Zeolites^a^

sample	cation/mmol g^–1^	*Q* (CO_2_)_sat._/mmol g^–1^	*Q* (CO_2_)/molecules uc^–1^	*Q* (CO_2_)/molecules cation^–1^
Li^+^-GME	Li^+^ 4.0, Na^+^ 0.7	6.3	9.4	1.3
K^+^-GME	K^+^ 4.1	3.5	6.0	1.0^b^

a Adsorbed amount of CO_2_ obtained via the analysis of CO_2_ adsorption isotherms using the Langmuir–Freundlich equation.

b The number of CO_2_ molecules per cation was calculated using six cations because CO_2_ molecules could not be adsorbed onto cations in the gme cage.

Here, the volumes of the straight channel and the gme cage were theoretically calculated on the basis of the geometric structure of GME to verify the contribution ratio of the channel and cage to the amount of CO_2_ adsorbed within the framework volume (Figure S19 and Table S2). The volumes of the straight channel and gme cage in a unit cell were determined as 365 and 725 Å^3^ (the volume of one gme cage containing a Na^+^ ion is approximately 361 Å^3^), respectively. The CO_2_ molecules interacted more strongly with the Na^+^ ions within the gme cage than with the gme cage itself. Furthermore, one straight channel in a given unit cell contributed six gme cages, and one gme cage was affected by three straight channels, indicating that the GME unit cell comprises practically one straight channel and one gme cage. Thus, the fraction of the volume of the gme cages to the total framework volume was 49.8%. Notably, the fraction of the amount of CO_2_ adsorbed in step 2 to the total adsorbed amount was 45.3%, suggesting that the CO_2_ adsorption sites in steps 1 and 2 were straight channels and gme cages, respectively. These results validate the estimation of the adsorption sites on the basis of the relationship between the framework volume and the adsorption capacity. CO_2_ molecules with intramolecular polarity affect the process of stepwise adsorption because of the interaction between the CO_2_ molecules and Na^+^ ions in the GME framework. Next, the effects of H_2_O guest molecules, which exhibit a high dipole moment,^60^ on the adsorption isotherms (Figure S20) of the ion-exchanged GME zeolites were confirmed to investigate the cation-gate-opening ability of interpolar molecules other than CO_2_. In contrast to the CO_2_ adsorption isotherm of the Na^+^-GME zeolite, the H_2_O adsorption isotherm did not exhibit stepwise adsorption behavior and demonstrated a H_2_O adsorption performance equivalent to that of the Li^+^-GME zeolite. The dynamic molecular diameter of H_2_O is 2.64 Å,^60^ which is smaller than that of CO_2_ (3.30 Å^60^). Therefore, H_2_O was adsorbed in the straight channels and gme cages at relatively low humidities despite the presence of Na^+^ ions near the 8RW, resulting in distinct adsorption isotherm profiles from those for CO_2_.

### 3.6 CO_2_ Adsorption Sites and Isosteric Heat of Adsorption in GME Zeolites

Furthermore, a CO_2_ temperature-programmed desorption (CO_2_-TPD) analysis (Figure S21) was performed to confirm the CO_2_ adsorption sites in the Li^+^-GME and Na^+^-GME zeolites. After CO_2_ adsorption under 2 and 101.3 kPa CO_2_ atmospheres at 298 K, He was flowed into the samples, which were placed in a capillary at 20 mL/min (STP) and 298 K, after which the temperature was increased from 298 K under He flow. The Li^+^-GME zeolite provided three types of CO_2_ adsorption sites in its framework (Figure S21a). The first peak of the CO_2_ desorption was attributed to physisorption in the GME framework. The second and third desorption peaks in the TPD profiles indicated strong interactions between the CO_2_ molecules and Li^+^ ions. The second and third peaks revealed particularly strong interactions with Li^+^ ions at the SII and SI’ sites, respectively, because the desorption of CO_2_ adsorbed on Li^+^ ions at the SI’ site within the gme cage was difficult compared to that of CO_2_ adsorbed on Li^+^ ions at the SII site in the straight channel. These peaks did not change with no dependence on the CO_2_ pressure. In other words, CO_2_ was strongly adsorbed on Li^+^ ions under low pressures. In contrast, the Na^+^-GME zeolite exhibited two CO_2_ adsorption sites. The first desorption peak within the 50–100 °C temperature range indicated CO_2_ physisorption in the GME framework, whereas the second desorption peak indicated strong interactions between CO_2_ molecules and Na^+^ ions at the SIII or SII site (Figure S21b). Interestingly, the second desorption at *P*_CO2_ = 101.3 kPa shifted to a temperature higher than that at *P*_CO2_ = 2.0 kPa, suggesting that the change in the Na^+^ position within the GME framework affected the magnitude of the interactions of CO_2_ with the Na^+^ ions.

The CO_2_ adsorption isotherms at 298 and 318 K (Figure S22a–c) were obtained and subsequently analyzed by using the L–F equation. The change in the isosteric adsorption enthalpy of the as-prepared and ion-exchanged GME zeolites was calculated by using the Clausius–Clapeyron equation (Figure S23). The changes in the enthalpy of the Li^+^- and K^+^-GME zeolites at *Q* = 0.01 mmol g^–1^ were determined as 75.4 and 57.4 kJ mol^–1^, respectively, indicating interactions between CO_2_ molecules and extra-framework cations. The changes in the enthalpy of the CO_2_ adsorption subsequently decreased with increasing CO_2_ uptake. This behavior has been previously reported in the literature.^1,61^ First, CO_2_ was adsorbed onto extra-framework cations in the pores, and then, CO_2_ was physisorbed in them. Additionally, the enthalpy change behavior of the Na^+^-GME zeolite differed from that of the Li^+^-GME and K^+^-GME zeolites. The change in enthalpy gradually increased to 2.5 mmol g^–1^, indicating that the CO_2_ molecules were adsorbed onto Na^+^ ions in the straight channels. A sharp decrease in the enthalpy change was subsequently observed between 2.5 and 2.9 mmol g^–1^. Finally, the change in enthalpy remained constant, suggesting that Na^+^ ions migrated between the cation sites. In other words, some of the heat of adsorption was substituted for the change in the potential energy of the Na^+^ ions with migration between the cation sites. In summary, the profiles of the heat of adsorption suggested that the heat of the CO_2_ adsorption in the Na^+^-GME zeolite compensated for the potential energy difference between the Na^+^ ion sites.

### 3.7 CO_2_ Adsorption Mechanisms of GME Zeolites

On the basis of the analysis and calculation results (Figure 6a,c), a CO_2_ adsorption mechanism for the GME zeolites was proposed (Figure 6b,d). The Li^+^ ions located at the SII sites do not obstruct the diffusion of guest molecules, such as CO_2_ and N_2_, into the gme cages. Therefore, CO_2_ molecules initially diffuse into the straight channels and then are adsorbed onto Li^+^ ions in both the straight channels and the gme cages within the Li^+^-GME zeolite. In contrast, K^+^ ions at the SIII sites block the entry of guest molecules into the gme cages, indicating that the CO_2_ molecules are adsorbed onto K^+^ ions in the straight channels of the K^+^-GME zeolite. In addition, these cations do not migrate in the GME framework, as verified by the fact that the PXRD patterns do not change during CO_2_ adsorption and desorption. In the case of the Na^+^-GME zeolite, CO_2_ molecules are adsorbed onto Na^+^ ions at the SIII sites. In other words, CO_2_ molecules are first adsorbed in the straight channels, resulting in a sharp increase in the level of CO_2_ uptake under low pressures in step 1. Afterward, Na^+^ ions gradually migrate from the SIII to SII sites, and then, CO_2_ molecules can be adsorbed in the gme cages, as indicated by the CO_2_ adsorption isotherm in step 2. Furthermore, the magnitude of the interaction between the CO_2_ and Na^+^ ions in the gme cages is greater than that between the CO_2_ and Na^+^ ions in the straight channels. Therefore, the CO_2_ desorption process of the Na^+^-GME zeolite results in a hysteresis loop due to the change in the potential energy profile of the GME framework after migration of Na^+^ in the straight channels.

**Figure 6:**
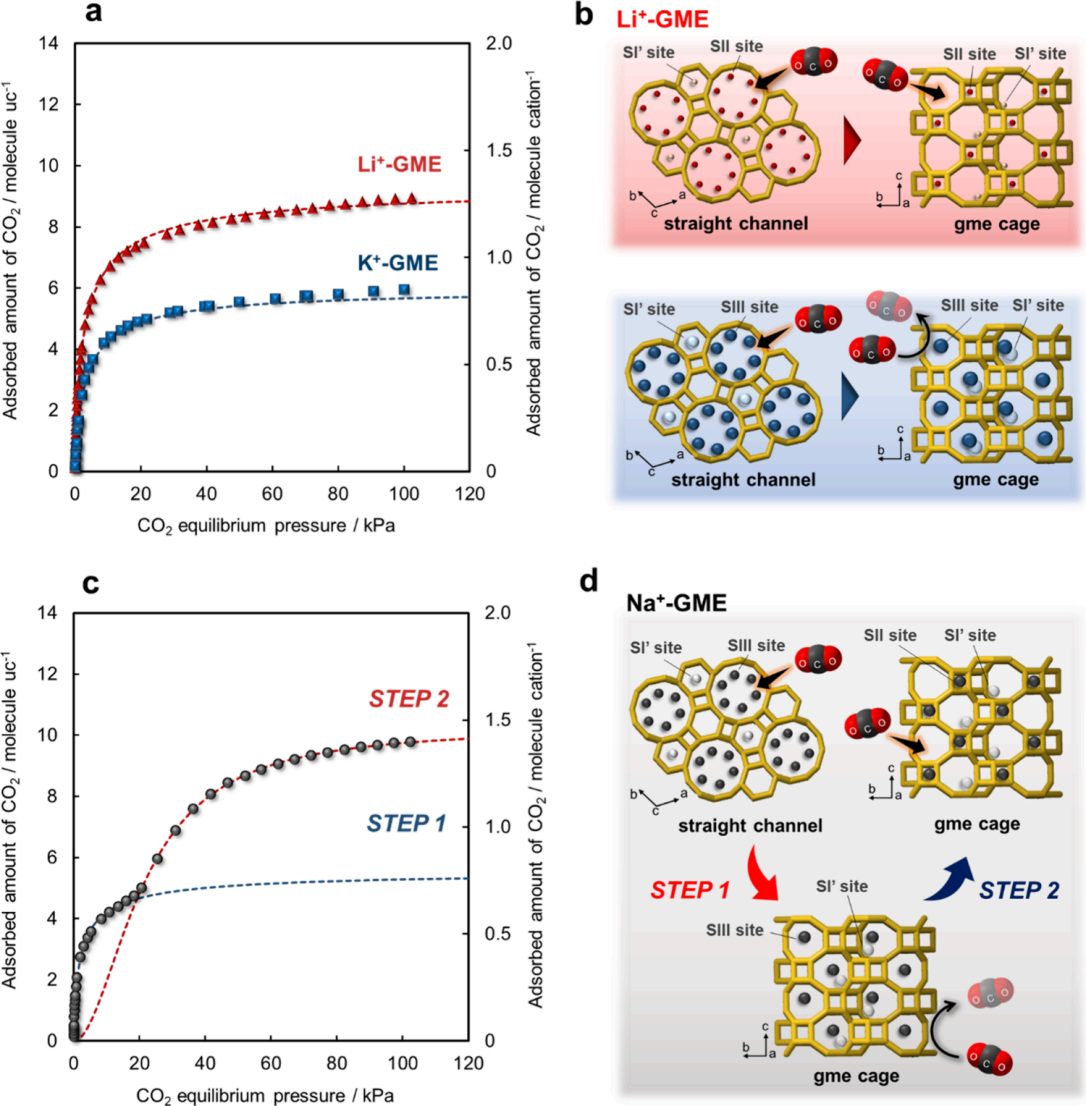
CO_2_ adsorption mechanisms of the GME zeolite. (a) CO_2_ adsorption isotherms of the (▲) Li^+^- and (■) K^+^-GME zeolites at 298 K. (b) CO_2_ adsorption mechanisms of the Li^+^-GME and K^+^-GME zeolites. (c) CO_2_ adsorption isotherm of the Na^+^-GME zeolite at 298 K. (d) CO_2_ adsorption mechanisms of the Na^+^-GME zeolite. The dashed lines are derived from curve fitting analysis using the L–F equation, as expressed in eq 2.

### 3.8 Effect of the Pelletization of the GME Zeolite Powder on CO_2_ Adsorption

Finally, the Na^+^-GME zeolite powder was pelletized via a molding process to investigate the effect of pelletization on CO_2_ stepwise adsorption (Figure 7a–d). Previous studies have revealed that the gate-adsorption behavior exhibited by pelletized flexible MOFs is reduced due to the loss of structural flexibility.^62^ Therefore, concerns have been raised that pelletization may influence the stepwise adsorption of the Na^+^-GME zeolite. However, the pelletized Na^+^-GME zeolite exhibited a stepwise adsorption analogous to that observed for the powder Na^+^-GME zeolite (Figure 7a). In the previous section, the CO_2_ stepwise adsorption by the Na^+^-GME zeolite was revealed to be derived not from structural flexibility but from cation migration in its pores. In other words, powder molding did not affect the cation migration phenomenon in zeolite pores. These results indicate that the Na^+^-GME zeolite exhibits the potential for use as a CO_2_ adsorbent in practical PSA processes.

**Figure 7:**
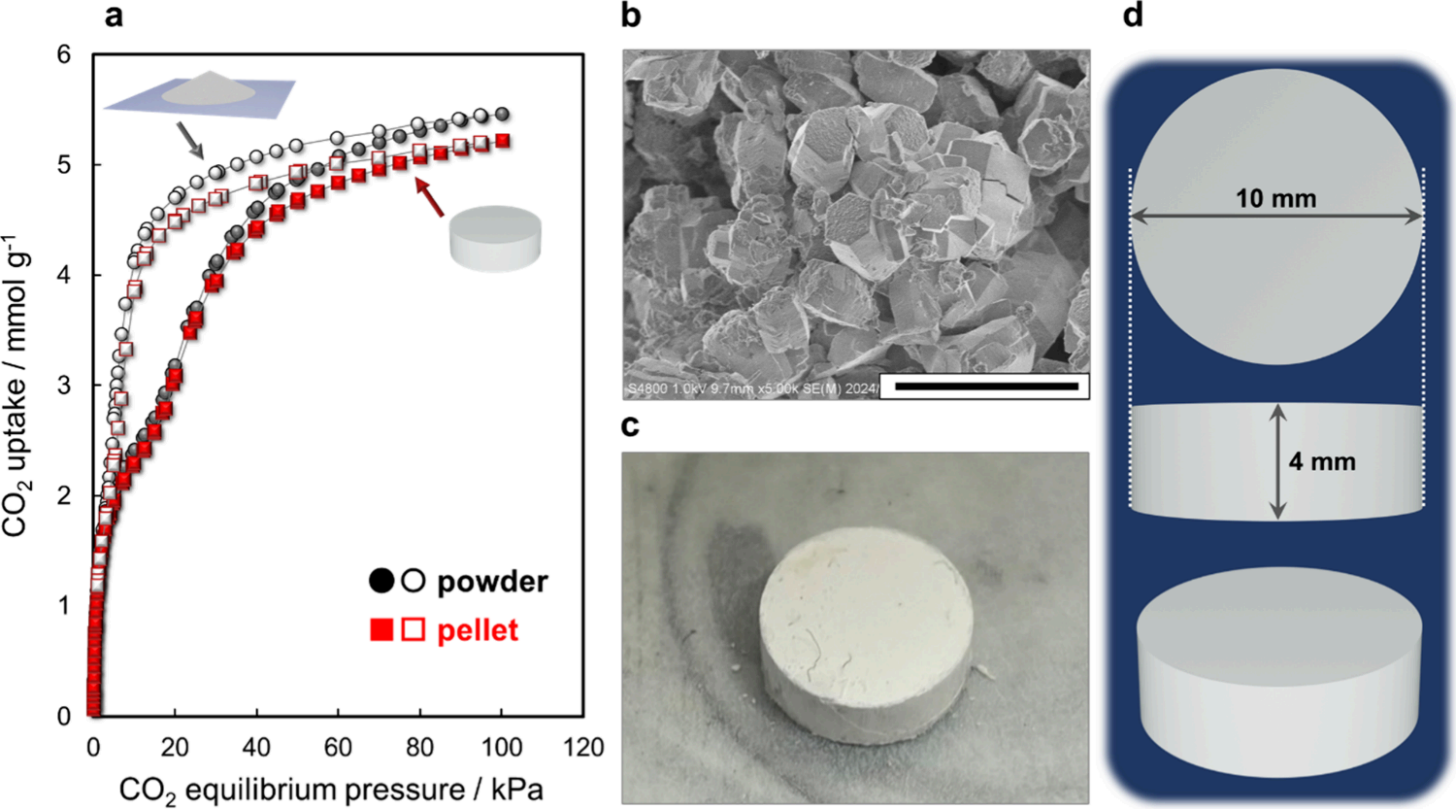
Effect of pelletization on the stepwise CO_2_ adsorption performance. (a) CO_2_ adsorption and desorption isotherms of the powdered and pelletized Na^+^-GME zeolites at 298 K. (b) FE-SEM image of Na^+^-GME zeolite particles in powder form (scale bar, 10.0 μm). (c) Image of the molded Na^+^-GME zeolite. (d) Illustration of the molded Na^+^-GME zeolite.

### 3.9 Application of Na^+^-GME Zeolite to the PSA Process

Finally, the working capacities of Na^+^-FAU, Na^+^-GME, and Li^+^-GME zeolites were calculated in the range of 5–80 kPa of CO_2_ pressure to evaluate the PSA performance of them (Figure S24). In the same pressure range, Na^+^-GME zeolite exhibited the highest CO_2_ working capacity (3.61 mmol g^–1^) than that of Na^+^-FAU with the same Si/Al ratio and Li^+^-GME zeolite. In other words, Na^+^-GME zeolite can achieve a large CO_2_ working capacity utilizing the two-step adsorption behavior compared with other zeolites. In the future target, control of the gate-opening and gate-closing pressure can pave the way for the utilization of Na^+^-GME zeolite in a wide pressure range in the PSA process.

## 4 Conclusions

In summary, this study revealed that the Na^+^-GME zeolite exhibited a high CO_2_ adsorption capacity and two-step CO_2_ gate-adsorption behavior. In contrast, N_2_ and CH_4_ were not adsorbed onto the Na^+^-GME zeolite, indicating a high selectivity for CO_2_. ^23^Na MAS NMR analysis was performed to determine the locations of the Na^+^ ions in the GME zeolite. Furthermore, in situ PXRD analysis and calculation of the volume of the void space in the GME framework and the amount of CO_2_ adsorbed per unit cell revealed that the migration of Na^+^ ions between cation sites induced CO_2_ adsorption in the gme cage, resulting in the two-step uptake of CO_2_ adsorbed in the Na^+^-GME zeolite. The potential energy for the migration of Na^+^ ions from the SIII to SII sites was compensated for by the change in the enthalpy of CO_2_ adsorption. First, CO_2_ was adsorbed onto Na^+^ ions in the straight channel of the Na^+^-GME zeolite, and then, Na^+^ ions migrated from the SIII to SII sites with increasing CO_2_ loading in the straight channel, thereby inducing the adsorption of CO_2_ in the gme cages. The calculation of the amount of CO_2_ adsorbed per unit cell also clarified the CO_2_ adsorption mechanisms of the Li^+^- and K^+^-GME zeolites. In the case of the Li^+^-GME zeolite, CO_2_ was adsorbed in the straight channels and gme cages in the initial stage. In contrast, CO_2_ was adsorbed only in the straight channels in the K^+^-GME zeolite.

Time-resolved in situ PXRD analysis demonstrated a high migration rate of Na^+^ ions in the GME framework, and that gate-adsorption behavior was maintained in the pelletized GME zeolite.

This study revealed that the Na^+^-GME zeolite exhibits CO_2_ gate-adsorption behavior and elucidated the mechanism underlying this gate-adsorption behavior. Furthermore, highly selective adsorption of CO_2_ onto Na^+^-GME zeolite exhibits the potential to separate CO_2_ from N_2_ and CH_4_. The observed behavior, such as the Na^+^ migration rate and the gate-adsorption behavior of the pelletized zeolite that connect this material to practical CO_2_ adsorption processes, paves the way for the design of efficient CO_2_ adsorption processes based on zeolites that exhibit gate-adsorption behavior.

## Supplementary Material



## Notes

Chemical and materials, synthesis, ion-exchange, analysis, molding methods, PXRD analysis (S1), ^29^Si DDMAS and ^27^Al MAS NMR spectra (S2), illustration of the calculation for ideal micropore volume of Li^+^-GME zeolite (S3), FE-SEM image of Na^+^-GME zeolite particles (S4), CO_2_ adsorption and desorption isotherms (*x* axis: logarithmic scale) (S5), CO_2_ adsorption performance and adsorbed amount of CO_2_ of Na^+^-FAU zeolite (S6, Table S1), Repeated CO_2_ adsorption measurements for Na^+^-GME zeolite (S7), CO_2_, N_2_, CH_4_ adsorption isotherms (S8), illustrations of N_2_ adsorption mechanisms (S9), illustration of cation sites (S10), ^23^Na MAS (S11, S14, S18) and ^13^C DDMAS (S14) NMR spectra, CO_2_ adsorption isotherms of Na^+^/K^+^-GME zeolites (S12), illustration of the interaction between Na^+^ at SIII site and N_2_ (S13), in situ PXRD patterns (S15, S16), adsorbed amount of CO_2_ per unit cell and cation (S17, Table S2, S3), illustration of relationship between the volume space of the void space in the GME zeolite and adsorbed amount of CO_2_ (S19), H_2_O adsorption isotherms (S20), CO_2_–TPD profiles (S21), CO_2_ adsorption isotherms at 318 K (S22), isosteric adsorption enthalpy change (S23), and CO_2_ working capacities (S24) (PDF)
