# Supplementary material for: Selective and Reversible Cation-Gating Adsorption Behavior in Gmelinite Zeolites for Efficient CO2 Separation
Source: ACS Appl Mater Interfaces. 2025 Nov 29;17(51):69795–806. doi: 10.1021/acsami.5c17473 (PMC12754748; doi:10.1021/acsami.5c17473)
Supplement: Supplementary file 1 [file am5c17473_si_001.pdf]

Supporting Information

Selective and Reversible Cation-Gating Adsorption Behavior in Gmelinite Zeolites for Efficient CO_2 Separation.

Yuto Higuchi[a,c*], Chihiro Yasuda[b], Yuna Suetsugu[a], Satoshi Inagaki[d] and Shunsuke Tanaka[a,c,e*]

[a]Department of Chemical, Energy and Environmental Engineering, Faculty of Environmental and Urban Engineering, Kansai University, 3-3-35 Yamate-cho, Suita-shi, Osaka 564-8680 JAPAN

[b]Graduate School of Science and Engineering, Kansai University, 3-3-35 Yamate-cho, Suita-shi, Osaka 564-8680 JAPAN

[c]Organization for Research and Development of Innovative Science and Technology (ORDIST), Kansai University, 3-3-35 Yamate-cho, Suita-shi, Osaka 564-8680 JAPAN

[d]Division of Materials Science and Chemical Engineering, Yokohama National University, 79-5 Tokiwadai, Hodogaya-ku, Yokohama 240-8501, Japan

[e]Carbon Neutrality Research Center (CNRC), Kansai University, 3-3-35 Yamate-cho, Suita-shi, Osaka 564-8680, Japan

* Corresponding author

Tel/Fax: +81-6-6368-0851; E-mail: yhiguchi@kansai-u.ac.jp (Y. Higuchi); E-mail: shun_tnk@kansai-u.ac.jp (S. Tanaka)

METHODS

Chemicals and Materials

H^+-type FAU zeolite (SiO_2/Al_2O_3 = 5.5, HSZ320HOA) as a starting material for the GME zeolites was purchased from Tosoh Co., Japan. Granular sodium hydroxide, with a purity exceeding 97%, as a mineralizer in the interzeolite transformation process from FAU to GME zeolites, was purchased from FUJIFILM Wako Pure Chemical Corporation. Lithium chloride, sodium chloride, and potassium chloride were purchased from FUJIFILM Wako Pure Chemical Corporation for use as cation-exchange agents for the as-prepared GME zeolites.

Interzeolite Transformation from H^+-Type FAU to Na^+-Type GME Zeolite

Three grams of the H^+-type FAU zeolite, 0.5 g (12.5 mmol) of sodium hydroxide, and five YTZ® balls 10 mm in diameter were placed in a 250 mL ceramic pot. They were milled at 150 rpm for 30 min in a Pulverisette 6 planetary mill (Fritsch Japan). The mixture was placed in a Teflon-lined stainless-steel autoclave along with 3.0 g (167.0 mmol) of H_2O. Afterwards, the autoclave was heated at a temperature of 403 K for 24 h with a supply of H_2O as the steam source. The resulting powder was subsequently washed with distilled water until the pH reached 7 and then dried at a temperature of 373 K under atmospheric conditions overnight.

Ion-Exchange of GME Zeolites

A total of 1.0 g of GME zeolite was placed in 100 mL of 1 M lithium chloride, sodium chloride, or potassium chloride aqueous solution. The solution was stirred at room temperature for 24 h. This stirring process was repeated to reach the maximum ion exchange ratio. Afterwards, the ion-exchanged GME zeolites were collected via centrifugation at 6,000 rpm and then dried at a temperature of 373 K under atmospheric conditions overnight.

Analysis Methods for Gas Adsorption Isotherms

The experimentally obtained CO_2 adsorption isotherms for the GME zeolites were analysed via the L–F equation (Eq. 2) to calculate the amount of CO_2 adsorbed per unit cell and cation.

$$Q = \frac{Q_{max} K P^n}{1+KP^n} \quad (2)$$

where Q is the equilibrium amount of CO_2 adsorbed, Q_{max} is the saturation capacity, K is the adsorption equilibrium constant, n is the Freundlich parameter, and P is the equilibrium pressure.

Calculation of the Change in Isosteric Adsorption Enthalpy (ΔH_{ad})

The change in isosteric adsorption enthalpy (ΔH_{ad}) was calculated using the Clausius–Clapeyron equation (Eq. 3) on the basis of CO_2 adsorption isotherms obtained at two distinct temperatures (298 and 318 K).

$$\frac{dlnP}{dT} = \frac{\Delta H_{ad}}{RT^2} \tag{3}$$

where P is the equilibrium pressure, T is the temperature, ΔH_{ad} is the change in isosteric adsorption enthalpy, and R is the ideal gas constant. ΔH_{ad} is a constant in the temperature region from 298 to 318 K.

Molding Method for Na^+-GME Zeolite Powder

Na^+-GME zeolite powder was placed in a molding apparatus (NT–50H, NPa System Corp., Japan), and the sample was compressed at 10 kPa under 373 K for 1 h. The dimensions of the pellet obtained through the molding process were 10 mm (diameter) × 4 mm (height).

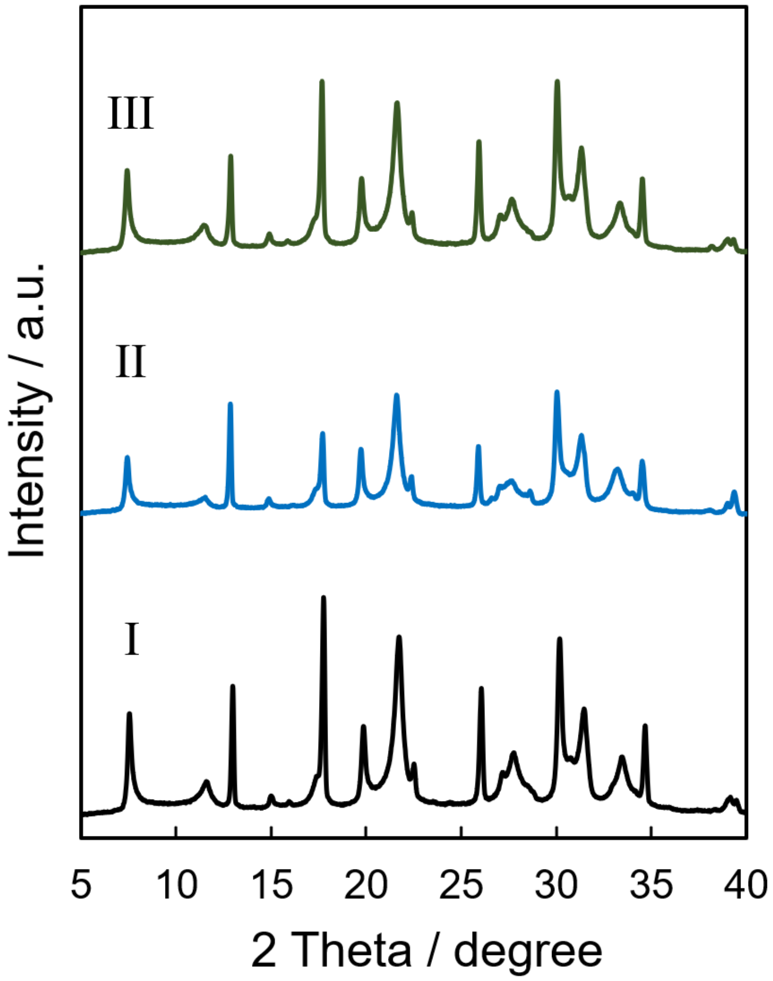

Figure S1. PXRD patterns of Na^+- and K^+-GME zeolites. (I) Na^+-GME zeolite. (II) K^+-GME zeolite. (III) Na^+-GME zeolite obtained by ion-exchange from K^+-GME zeolite (II).

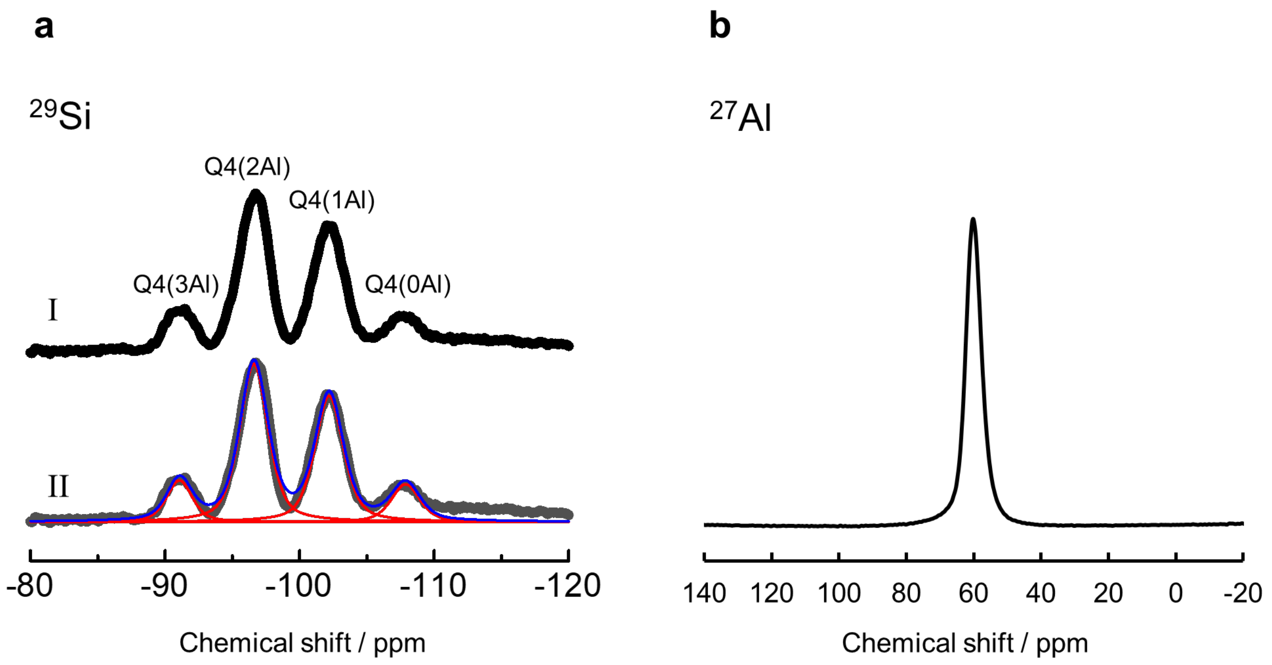

Figure S2. (a) (I) ^{29}Si DDMAS NMR spectrum of the as-prepared Na^+-GME zeolite. (II) Peak fitting curves for the NMR spectrum. (b) ^{27}Al MAS NMR spectrum of the as-prepared Na^+-GME zeolite.

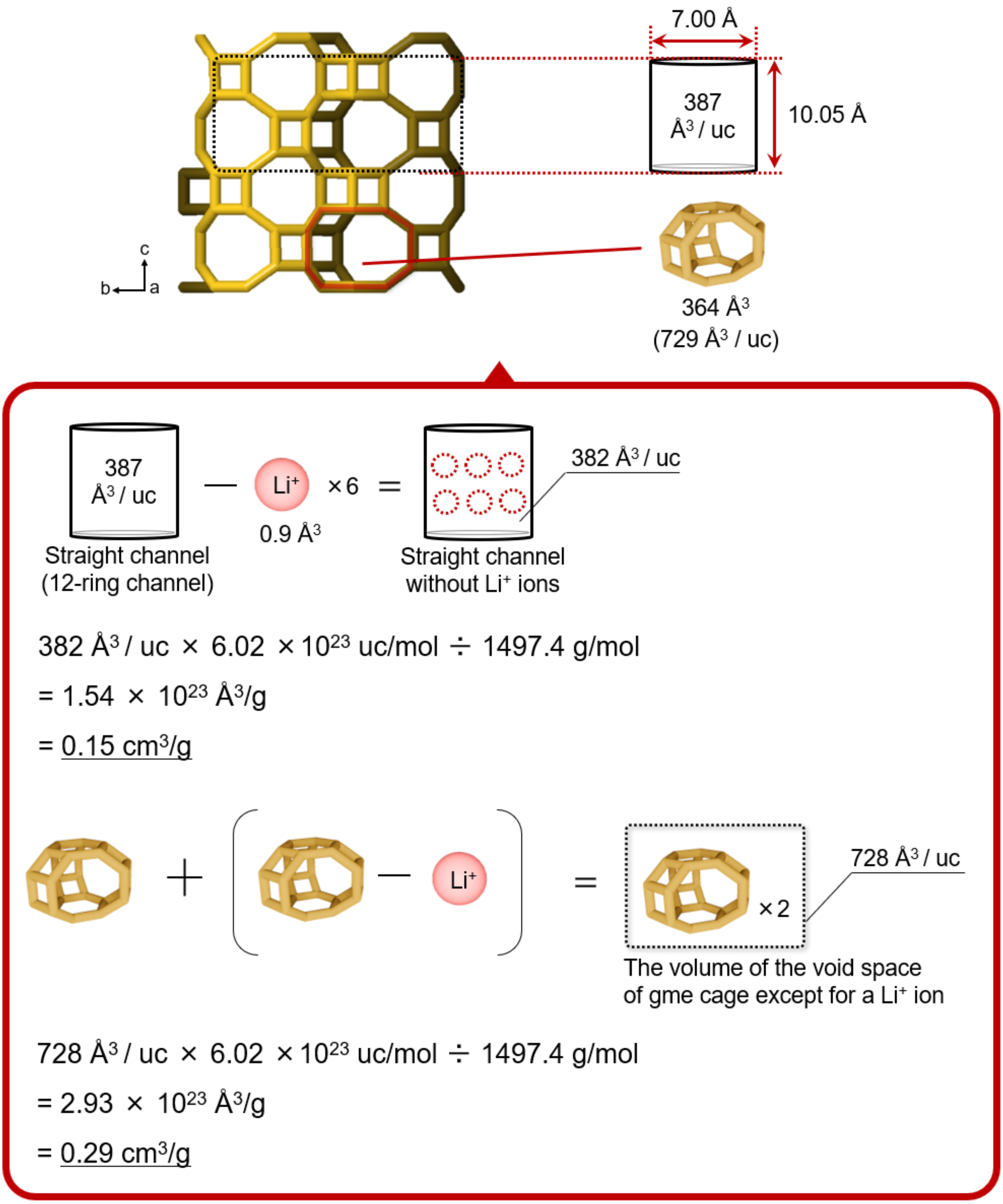

Figure S3. Calculation method of the ideal micropore volume of Li^+-GME zeolite based on the volume of the void space in the GME framework (ionic radius of Li^+: 0.60 Å [56]).

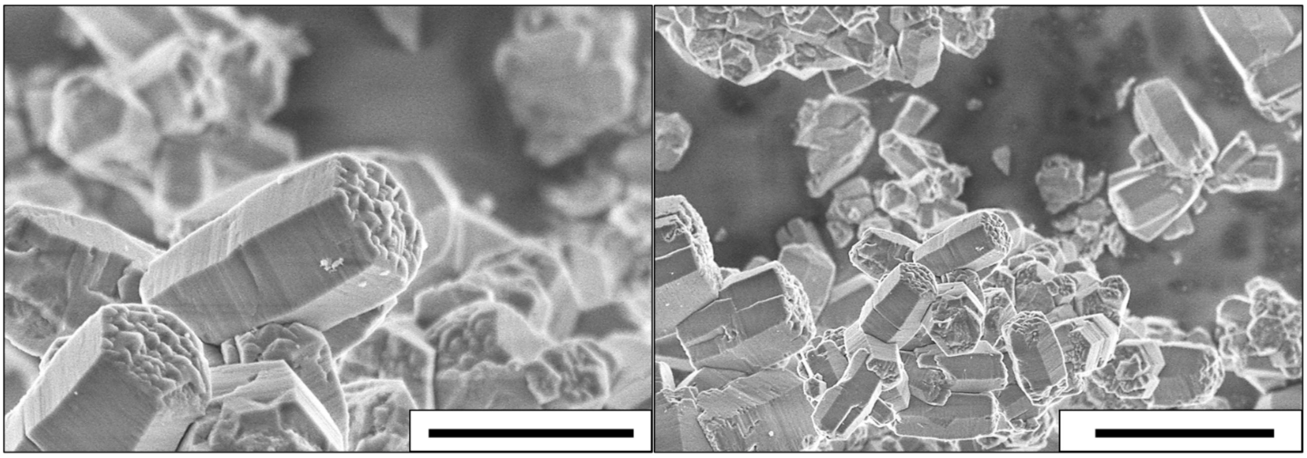

Figure S4. FE-SEM images of the as-prepared Na^+-GME (scale bars, left: 2.00 μm, right: 5.00 μm).

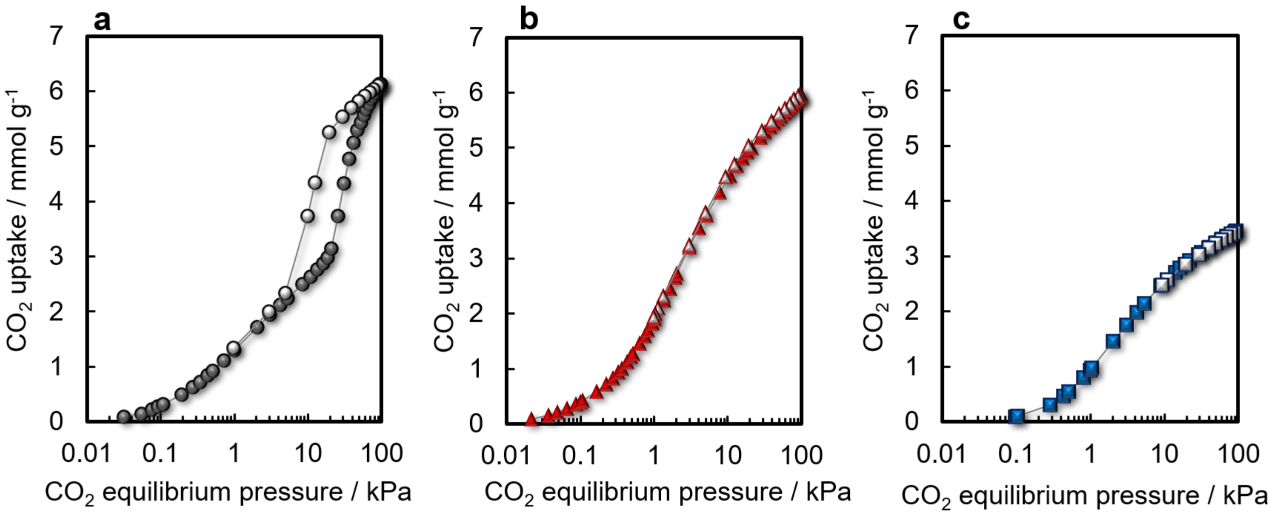

Figure S5. CO_2 adsorption and desorption isotherms at 298 K (*x* axis: logarithmic scale).
(a) As-prepared Na^+-GME, (b) Li^+-GME, and (c) K^+-GME zeolites (closed symbol: adsorption; open symbol: desorption).

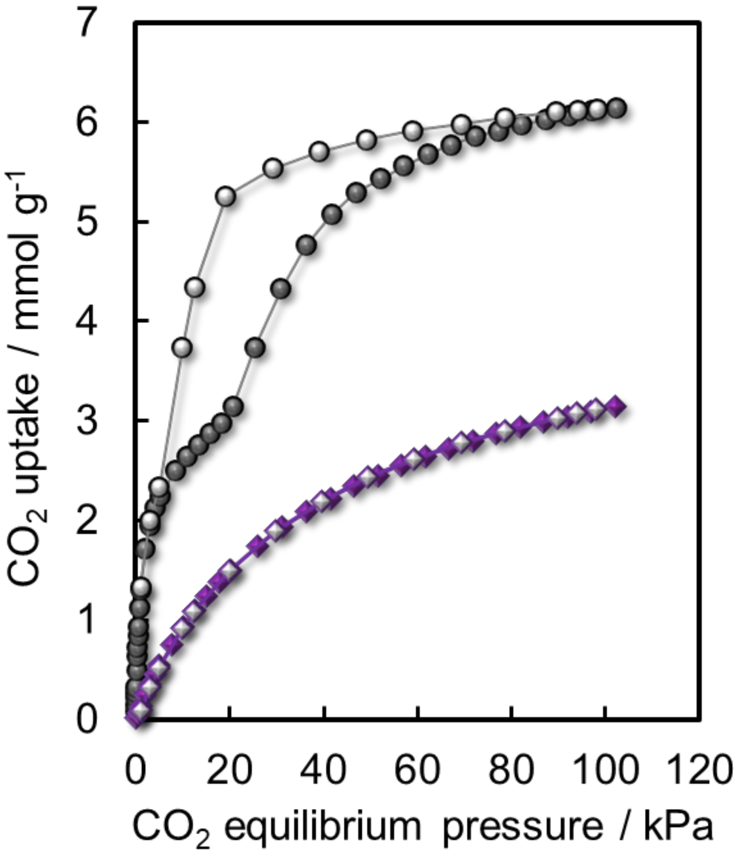

Figure S6. CO_2 adsorption and desorption isotherms of (●) Na^+-GME and (◆) Na^+-FAU at 298 K (closed symbols: adsorption; open symbols: desorption).

Table S1. Pore characteristics and Langmuir–Freundlich parameters for Na^+-FAU and Na^+-GME determined using CO_2 adsorption isotherms at 298 K.

Sample	Si/Al *1	S_{BET} / m^2 g^{-1} *2	V_{micro} / cm^3 g^{-1} *3	$Q(CO_2)_{sat.}$/ mmol g^{-1} *4	K *4	n *4
Na^+-FAU	2.69	1,132	0.44	4.5	0.030	0.94
Na^+-GME	2.42	66	0.025	6.4	0.0018	2.2

*1 Si/Al ratio calculated using ^{29}Si DDMAS NMR spectra.

*2 BET area calculated using N_2 adsorption isotherms at 77 K.

*3 Micropore volume calculated using the *t*-plot method and N_2 adsorption isotherms at 77 K.

*4 CO_2 saturated capacity and L-F parameters determined via L-F model fitting of CO_2 adsorption isotherms at 298 K.

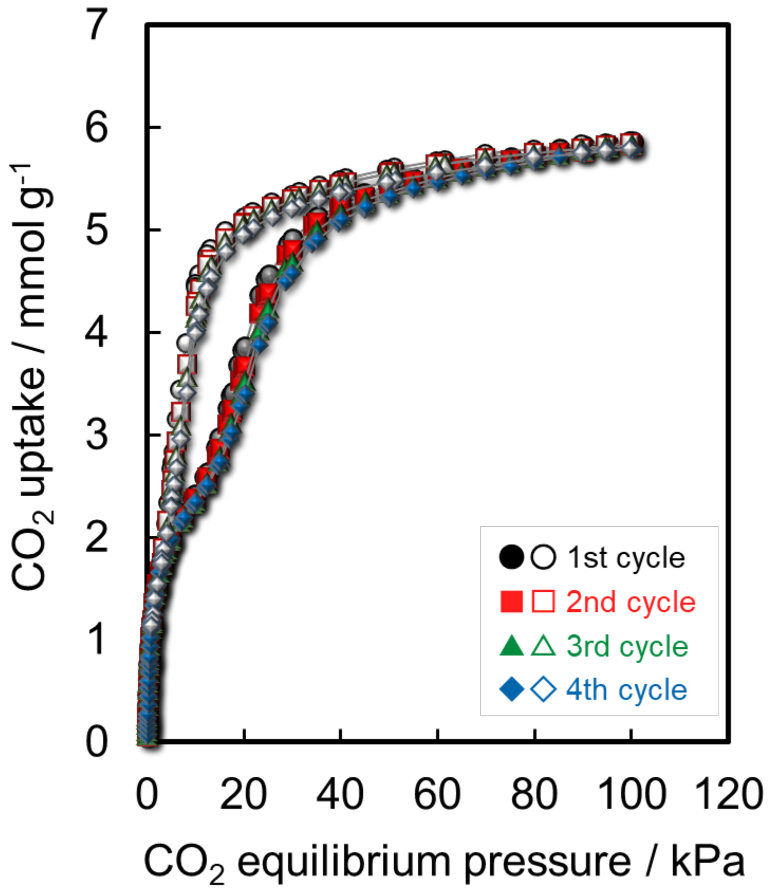

Figure S7. Repeated CO_2 adsorption measurements of Na^+-GME at 298 K (closed symbols: adsorption; open symbols: desorption).

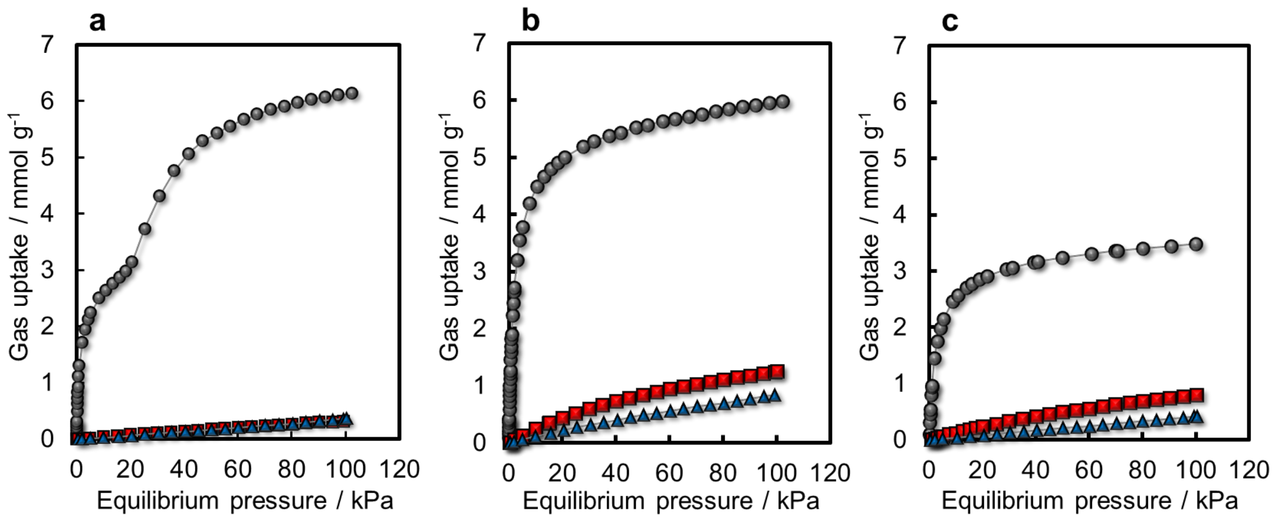

Figure S8. (●) CO_2, (▲) N_2, and (■) CH_4 adsorption isotherms at 298 K. (a) Na^+-GME, (b) Li^+-GME, and (c) K^+-GME zeolites.

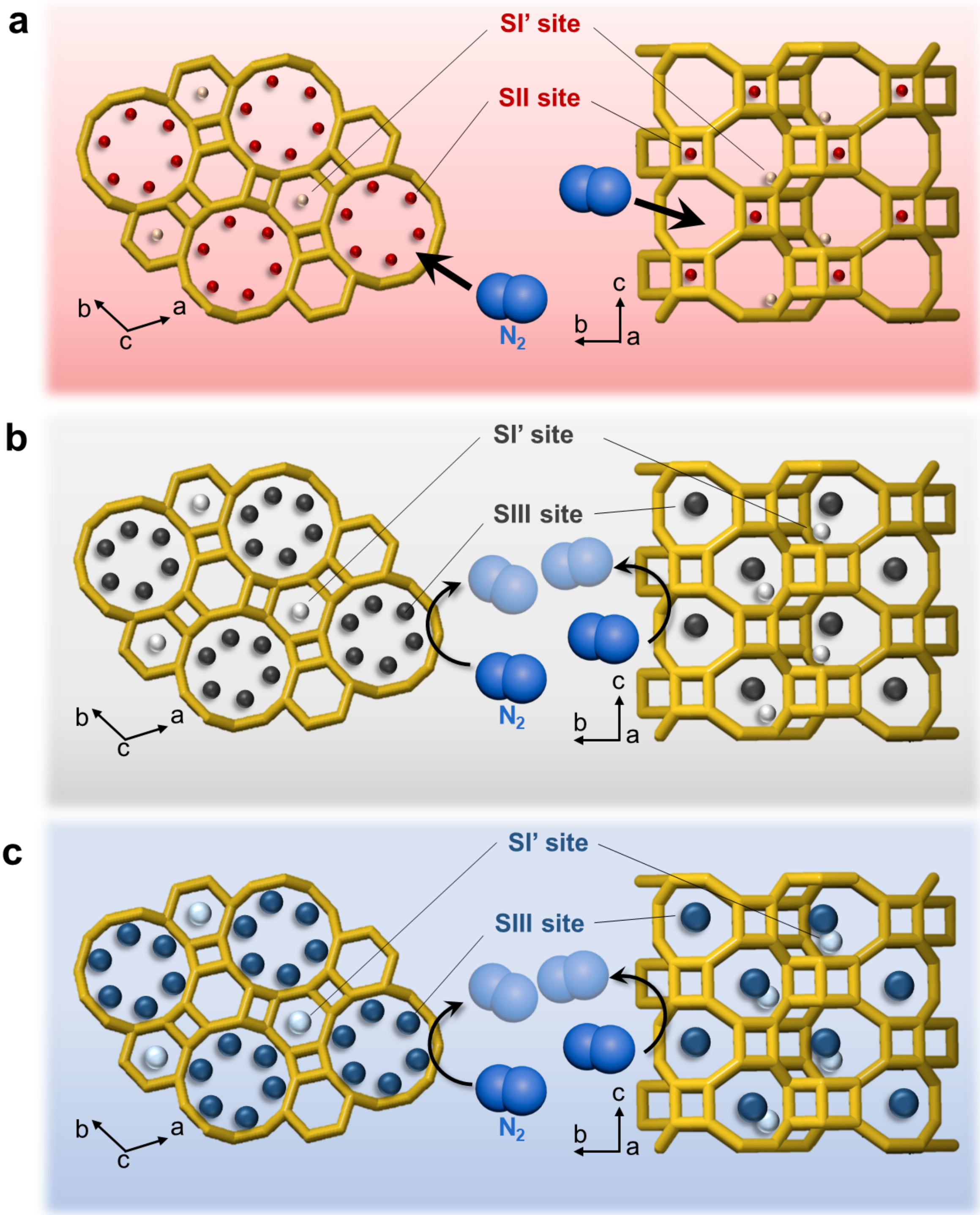

Figure S9. Plausible N_2 adsorption mechanism and estimation of each cation site on the basis of the N_2 adsorption behavior.
(a) Li^+-GME zeolite. (b) Na^+-GME zeolite. (c) K^+-GME zeolite.

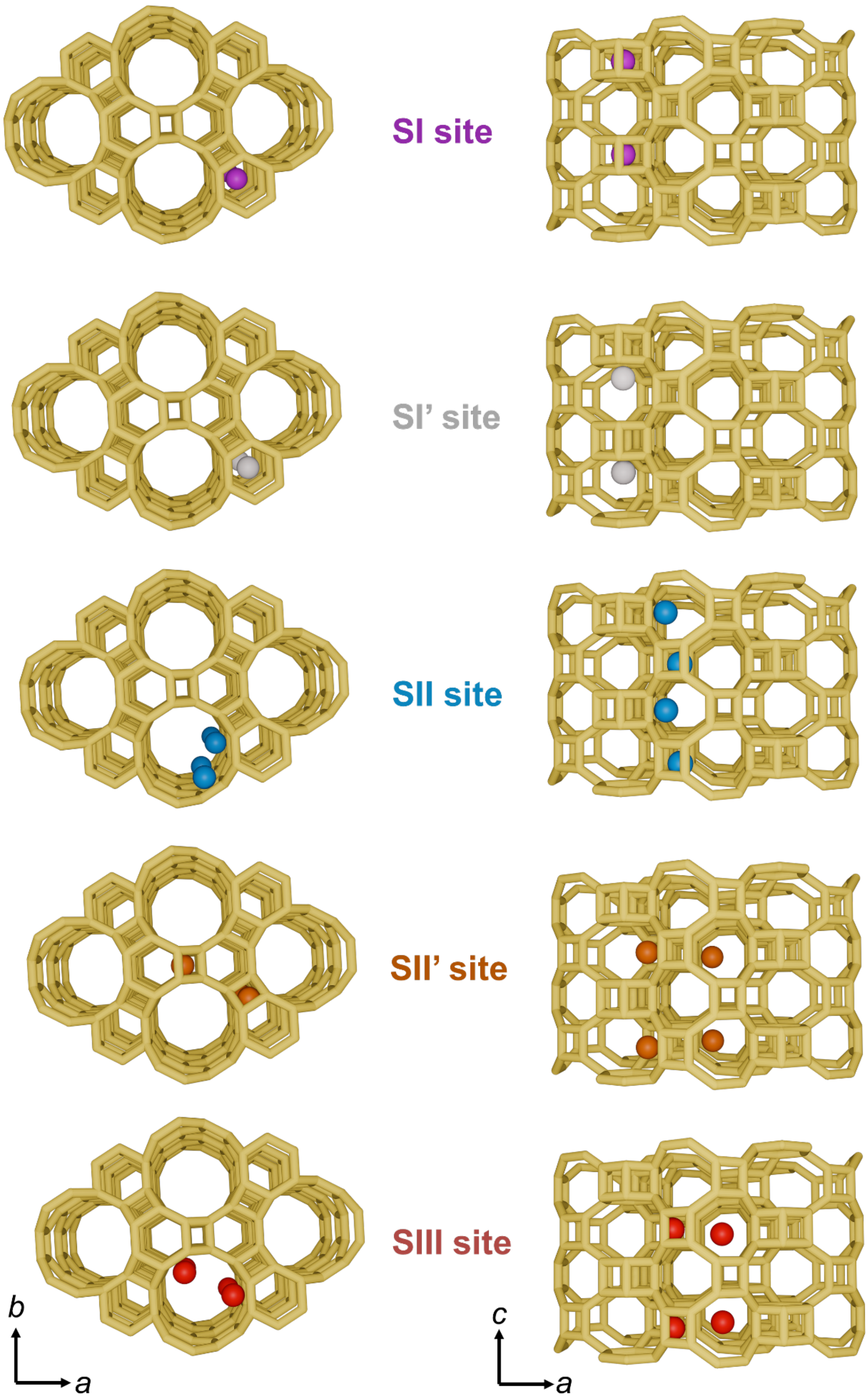

Figure S10. Cation sites in GME zeolites from the *b* and *c* axis viewpoints.

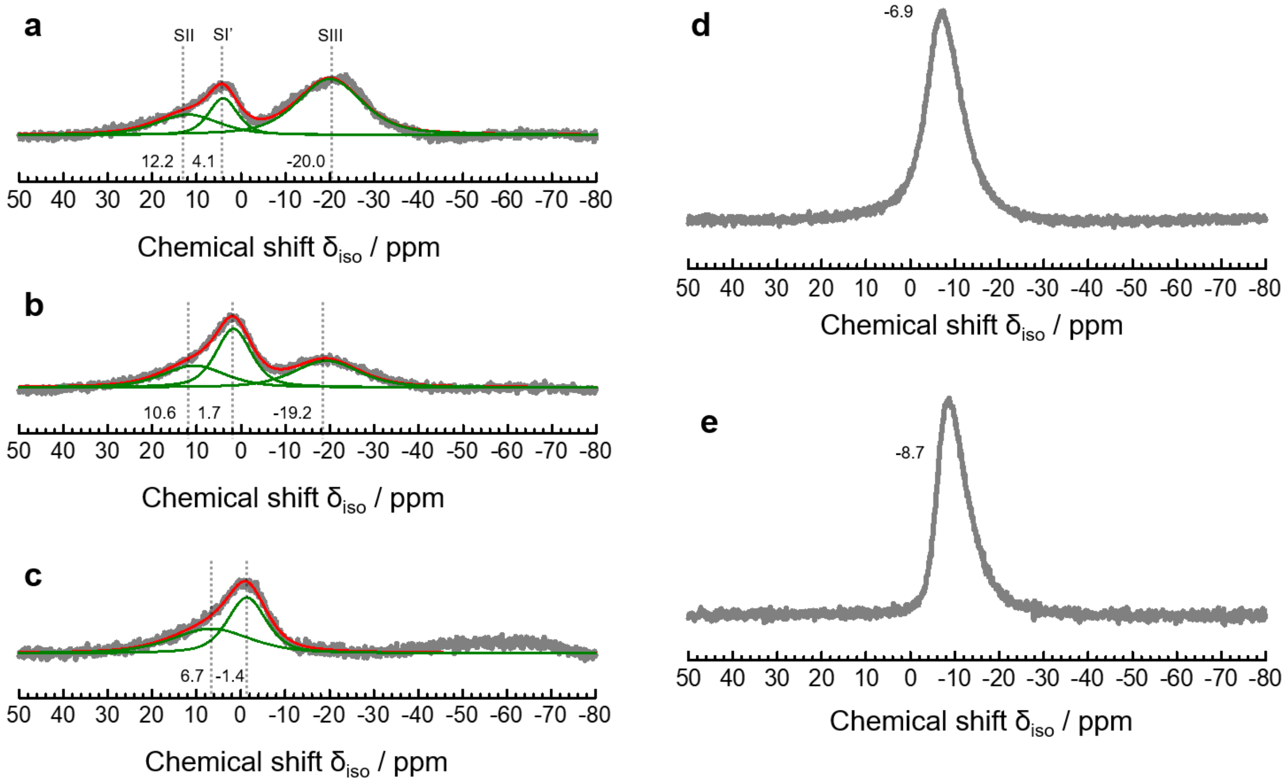

Figure S11. ^{23}Na MAS NMR spectra of dehydrated Na^+/K^+-GME under an Ar atmosphere. (a) Na^+: 100%. (b) Na^+: 88.9%, K^+: 11.1%. (c) Na^+: 70.9%, K^+: 29.1%. (d) Na^+: 48.7%, K^+: 51.3%. (e) Na^+: 5.9%, K^+: 94.1%.

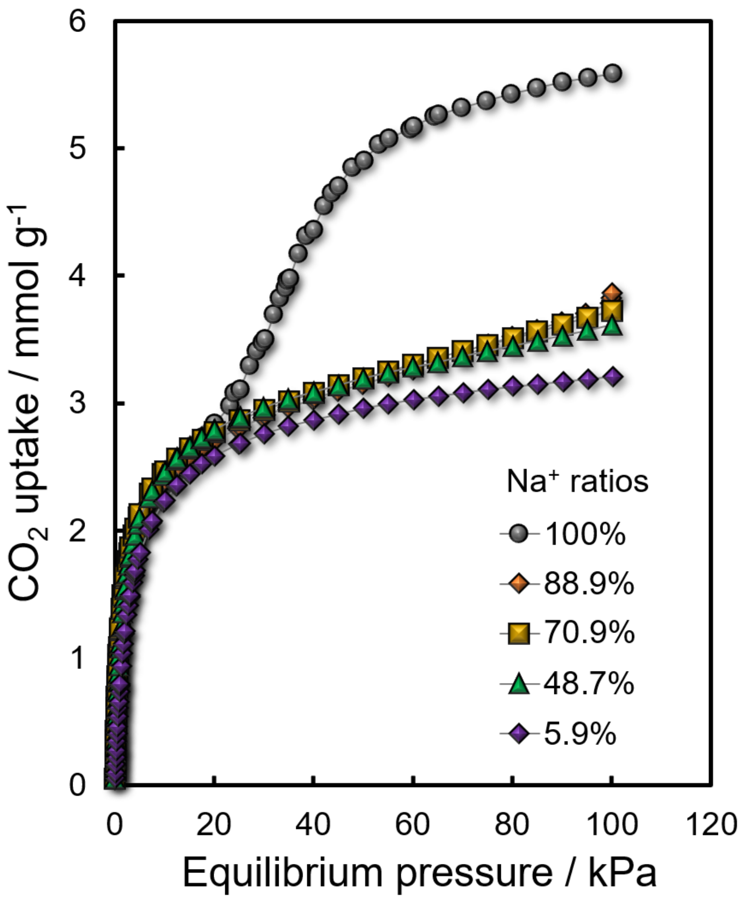

Figure S12. CO_2 adsorption isotherms of dual-cation-containing GME materials with various Na^+ and K^+ ion ratios at 298 K.

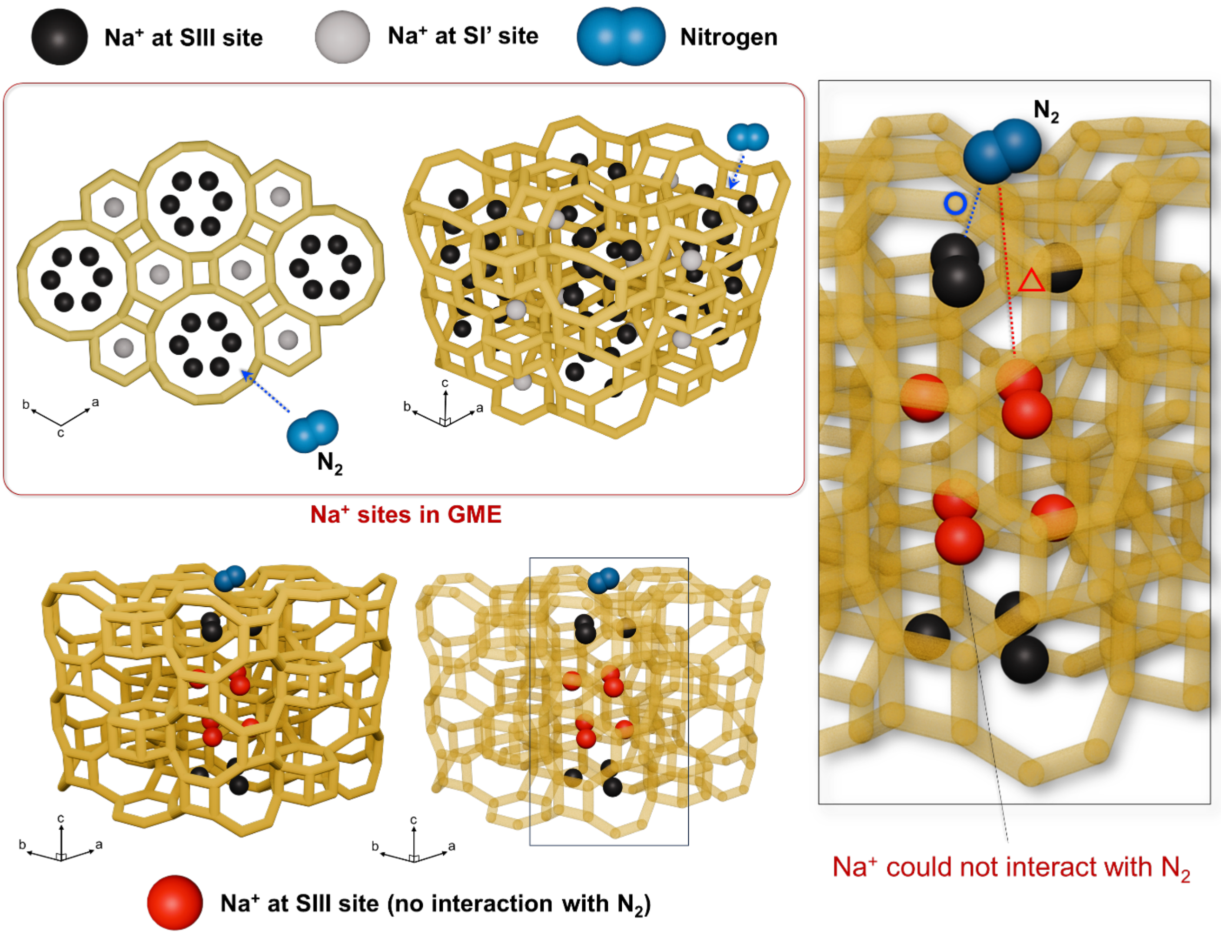

Figure S13. Na^+ at SIII sites in Na^+-GME accessible and inaccessible to N_2 molecules at 298 K.

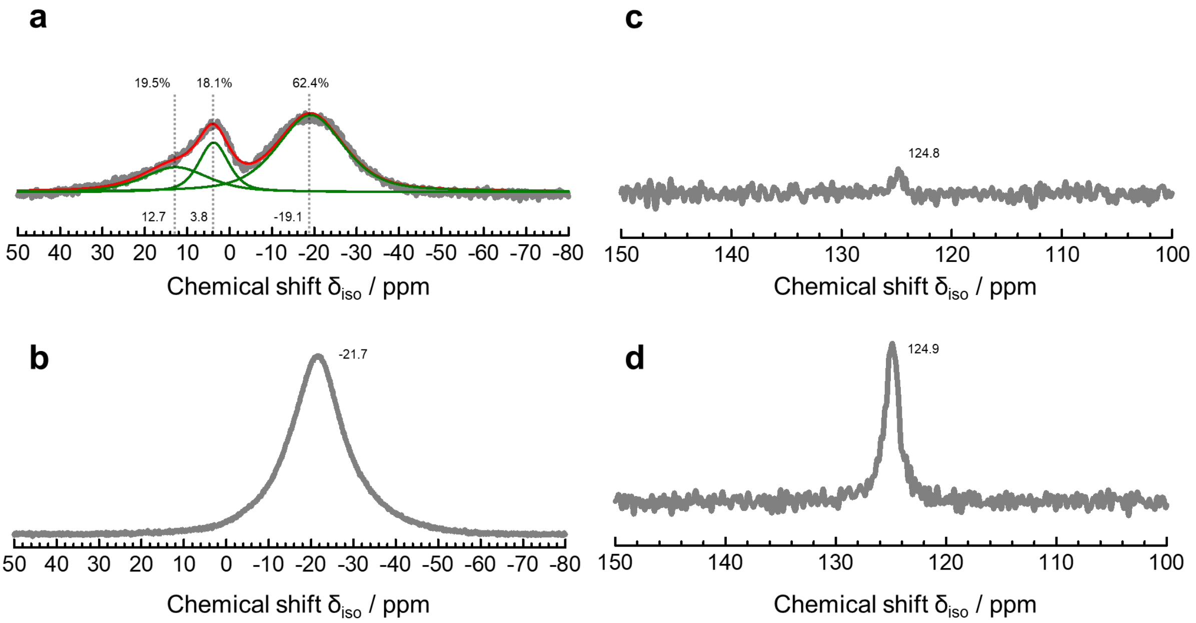

Figure S14. NMR spectra of Na^+-GME under a CO_2 atmosphere.
(a)(b) ^{23}Na MAS. (c)(d) ^{13}C DDMAS. (a)(c) 5% CO_2/Ar at 100 kPa. (b)(d) 100% CO_2 at 100 kPa.

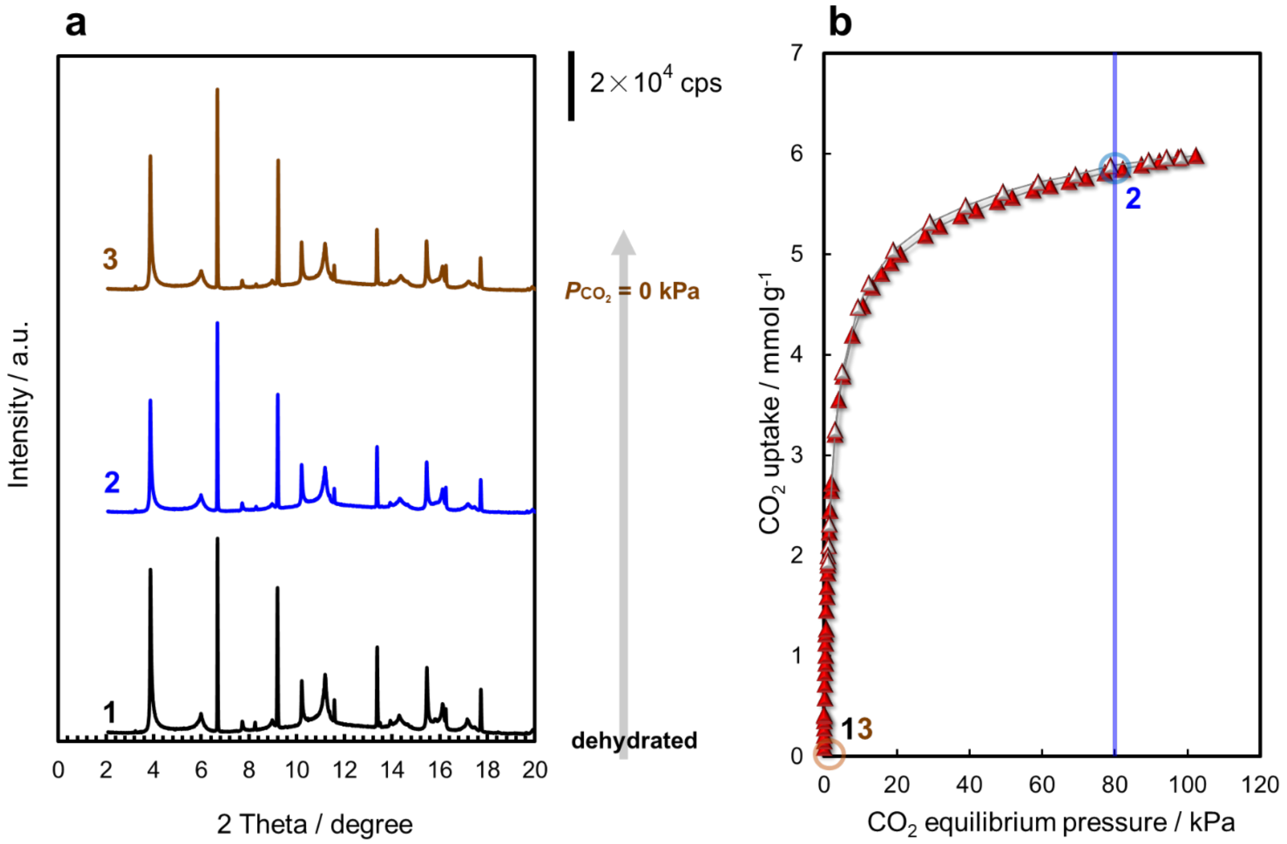

Figure S15. In situ PXRD analysis and CO_2 adsorption and desorption isotherms of the Li^+-GME zeolite at 298 K.

(a) In situ PXRD patterns during the CO_2 adsorption/desorption process (λ = 0.0799 nm). (b) CO_2 adsorption and desorption isotherms.

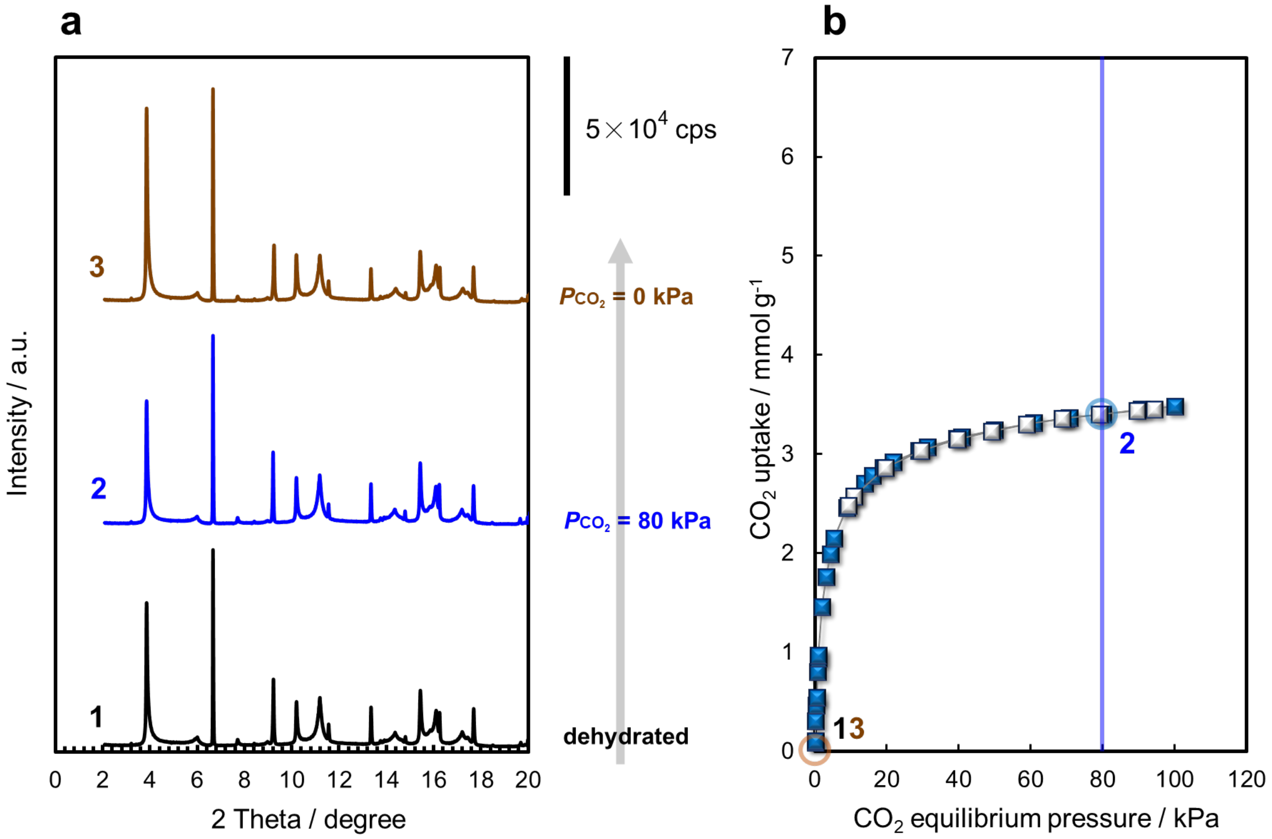

Figure S16. In situ PXRD analysis and CO_2 adsorption and desorption isotherms of the K^+-GME zeolite at 298 K.

(a) In situ PXRD patterns during the CO_2 adsorption/desorption process (λ = 0.0799 nm). (b) CO_2 adsorption and desorption isotherms.

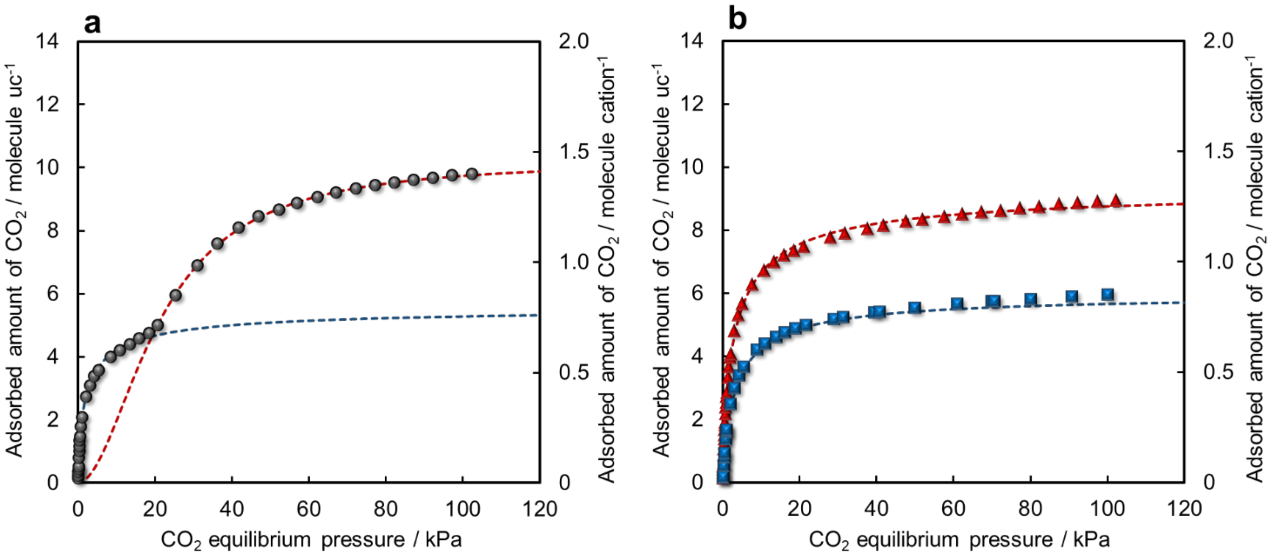

Figure S17. Number of adsorbed CO_2 molecules per unit cell and cation at 298 K.
(a) Na^+-GME. (b) (▲) Li^+- and (■) K^+-GME. The dashed lines are based on curve fitting analysis using the L-F equation in Eq. 2.

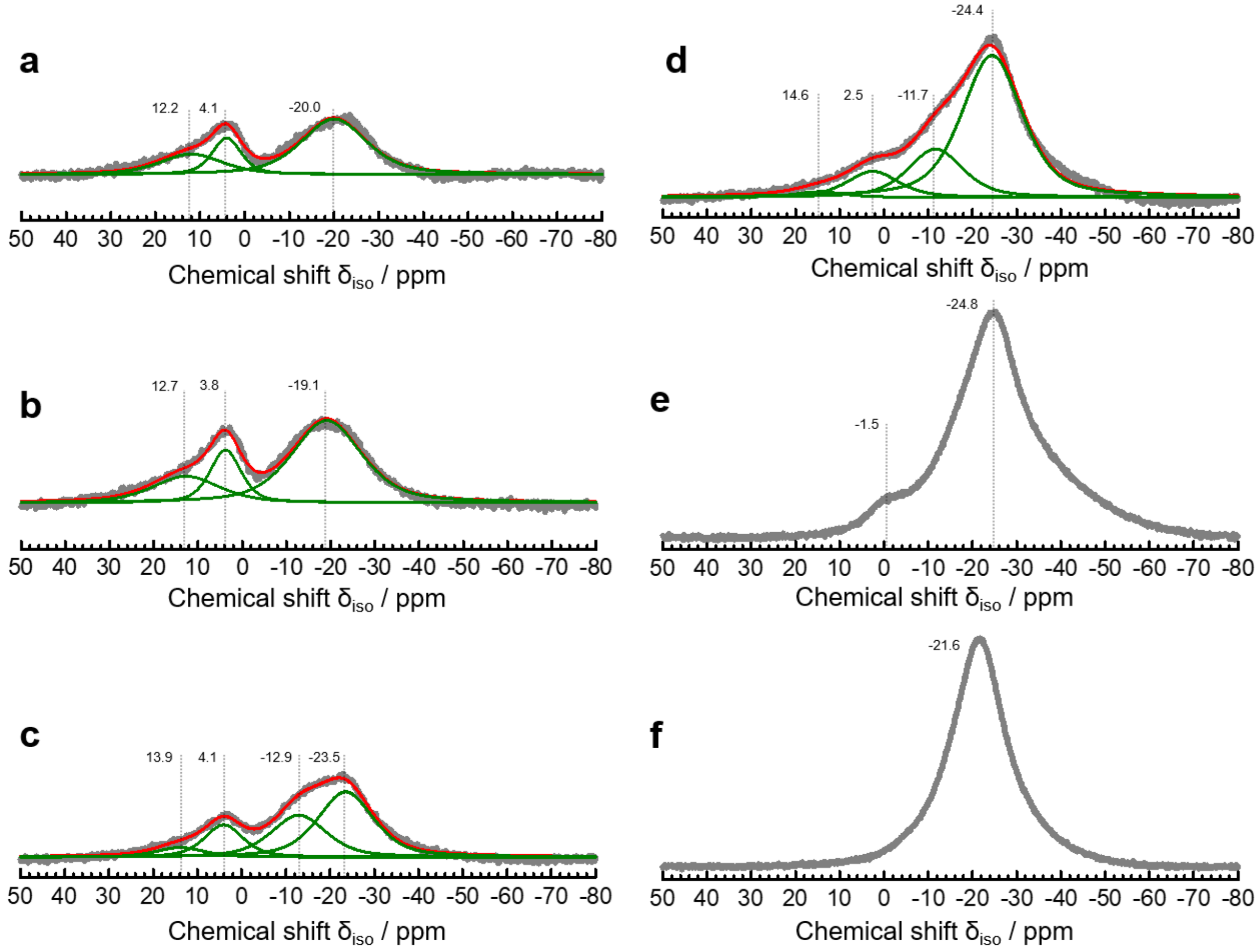

Figure S18. ^{23}Na MAS NMR spectra of Na^+-GME under a CO_2 atmosphere with arbitrary CO_2 pressure balanced using Ar at 100 kPa of total pressure.

(a) Ar (b) 5%CO_2/Ar (c) 15%CO_2/Ar (d) 30%CO_2/Ar (e) 50%CO_2/Ar (f) CO_2

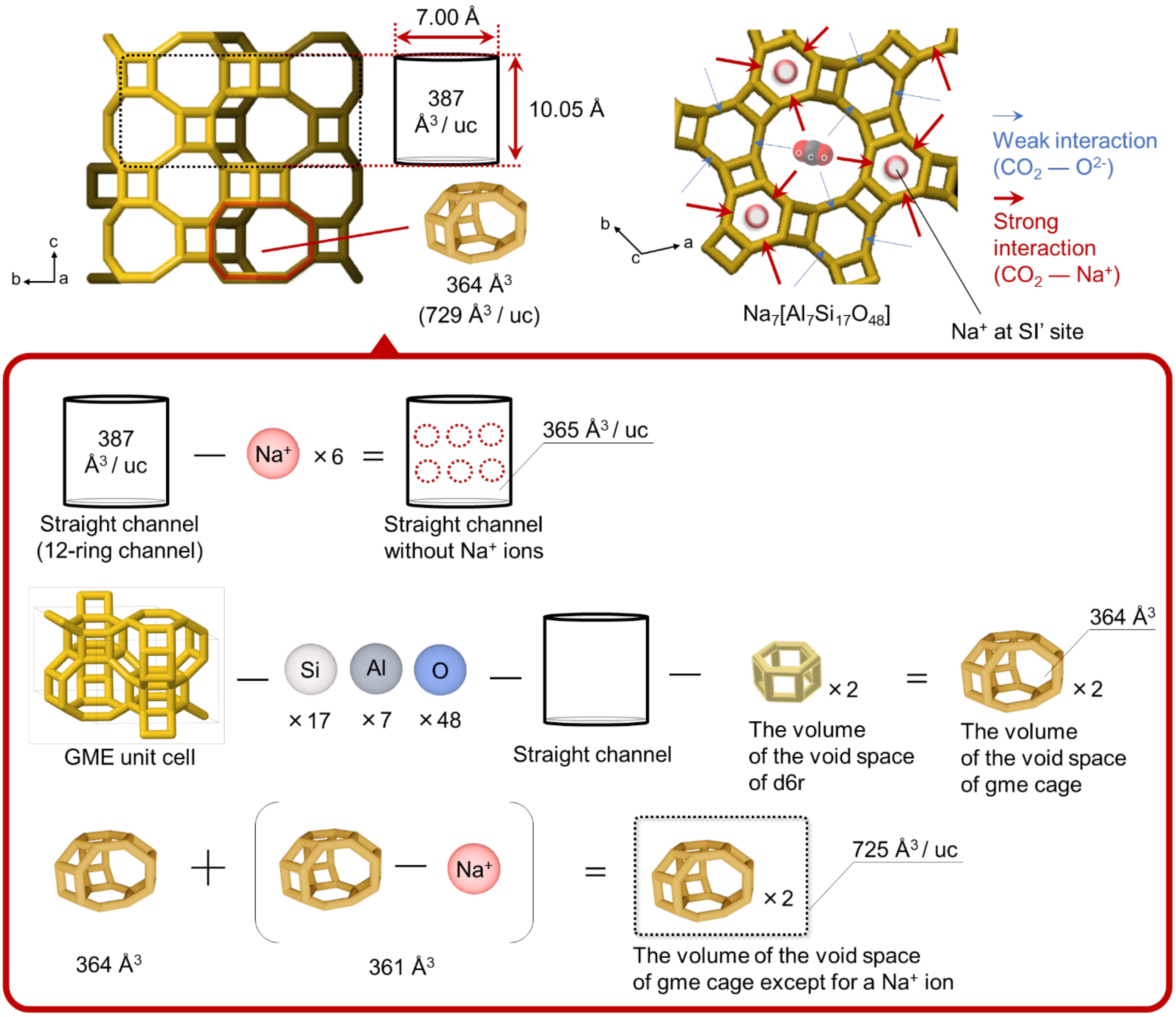

Figure S19. Relationship between the volume of the void space in the GME framework and the ratio of the amount of CO_2 adsorbed in the gme cage to the total adsorbed amount (ionic radius of Na^+: 0.95 Å [56]).

Table S2. Volumes of the gme cage and 12-ring channel of the GME unit cell.

	Ideal volume /Å^3 uc^{-1}	Volume of the void space except for Na^+ ions *1 /Å^3 uc^{-1}
gme cage (×2)	729	725
12-ring channel	387	365

*1 Six Na^+ ions were placed at SIII sites, and the other ion was placed at an SI' site.

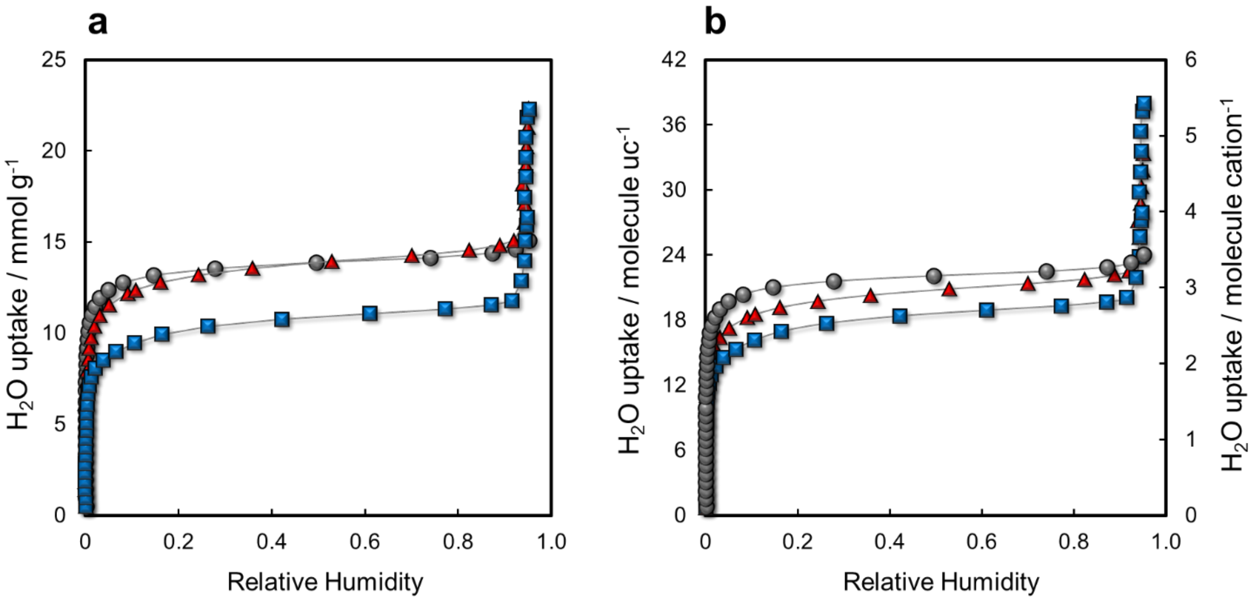

Figure S20. H_2O adsorption isotherms of (●) Na^+-, (▲) Li^+-, and (■) K^+-GME zeolites at 298 K. (a) mmol g^{-1}. (b) molecule uc^{-1} or $cation^{-1}$.

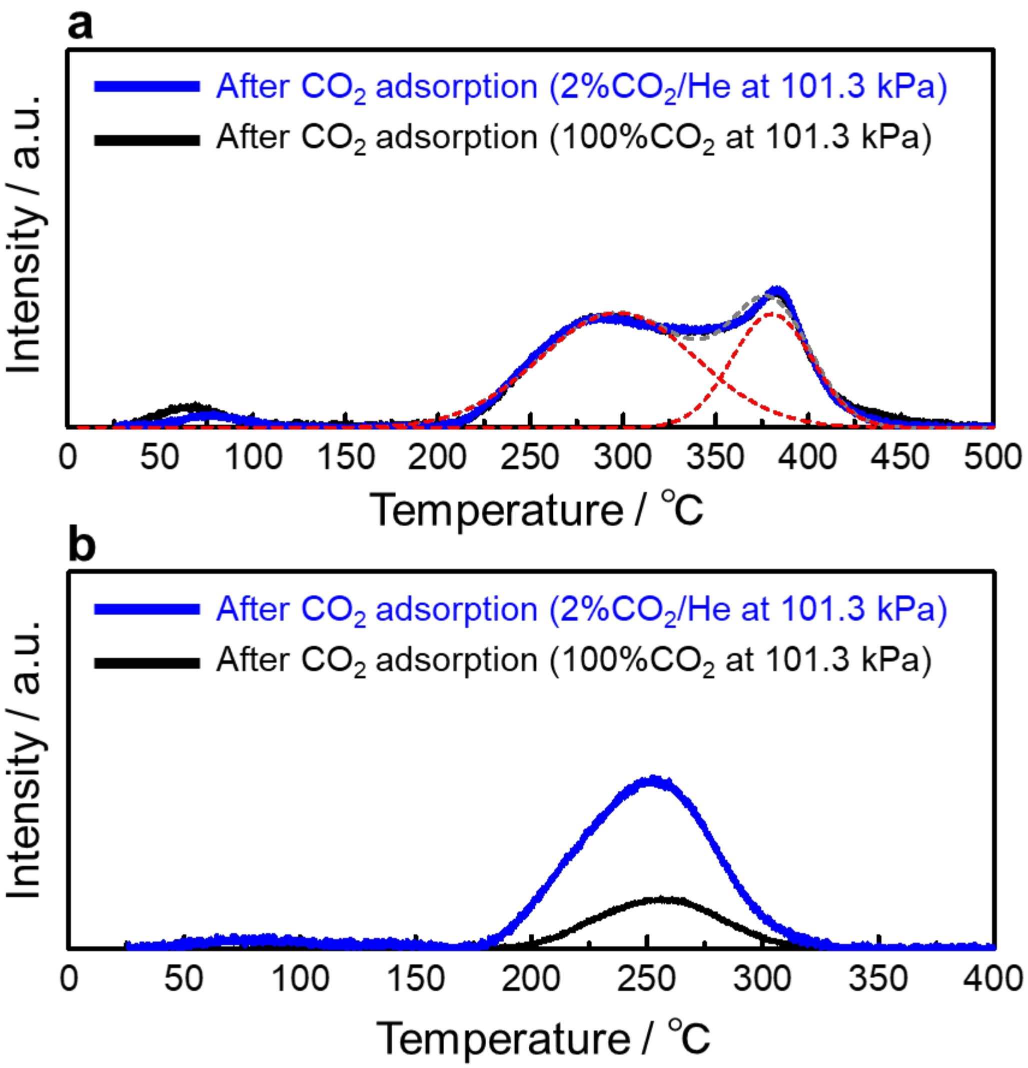

Figure S21. CO_2-TPD profiles (heating rate: 2 K/min, carrier gas: He 20 mL/min (STP)). (a) Li^+-GME zeolite. (b) Na^+-GME zeolite.

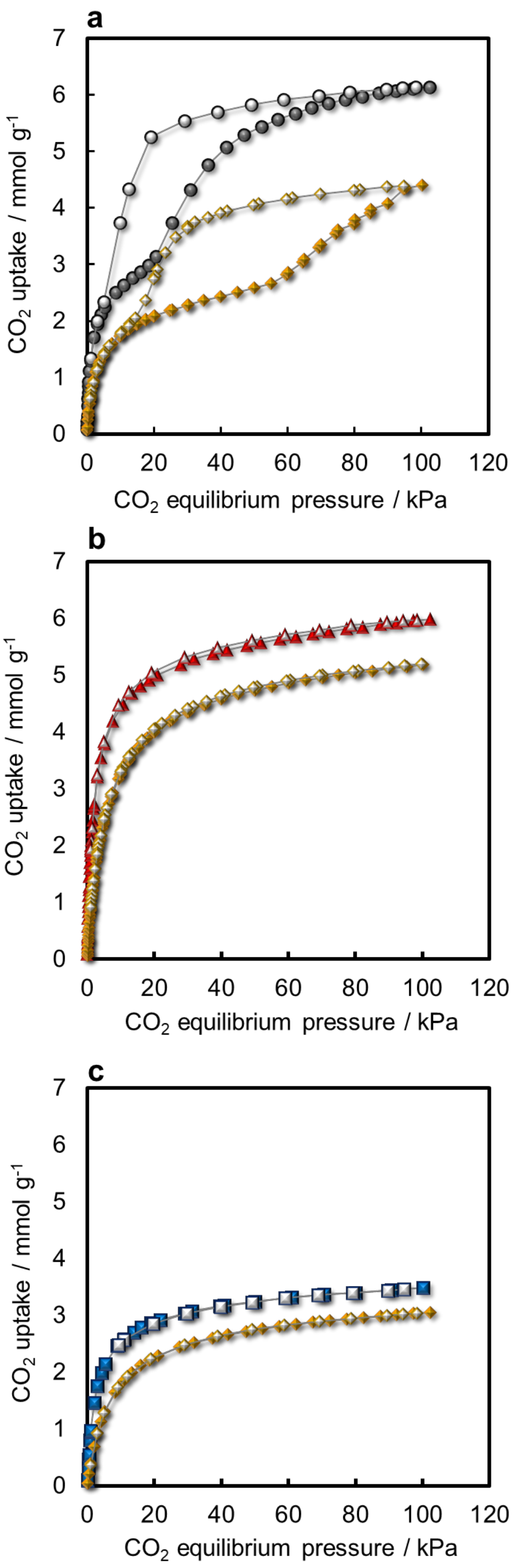

Figure S22. Effect of the adsorption temperature on the CO_2 adsorption performance.
(a) Na^+-GME zeolite. (b) Li^+-GME zeolite. (c) K^+-GME zeolite. (●)(▲)(■) 298 K, (◆) 318 K (closed symbols: adsorption; open symbols: desorption).

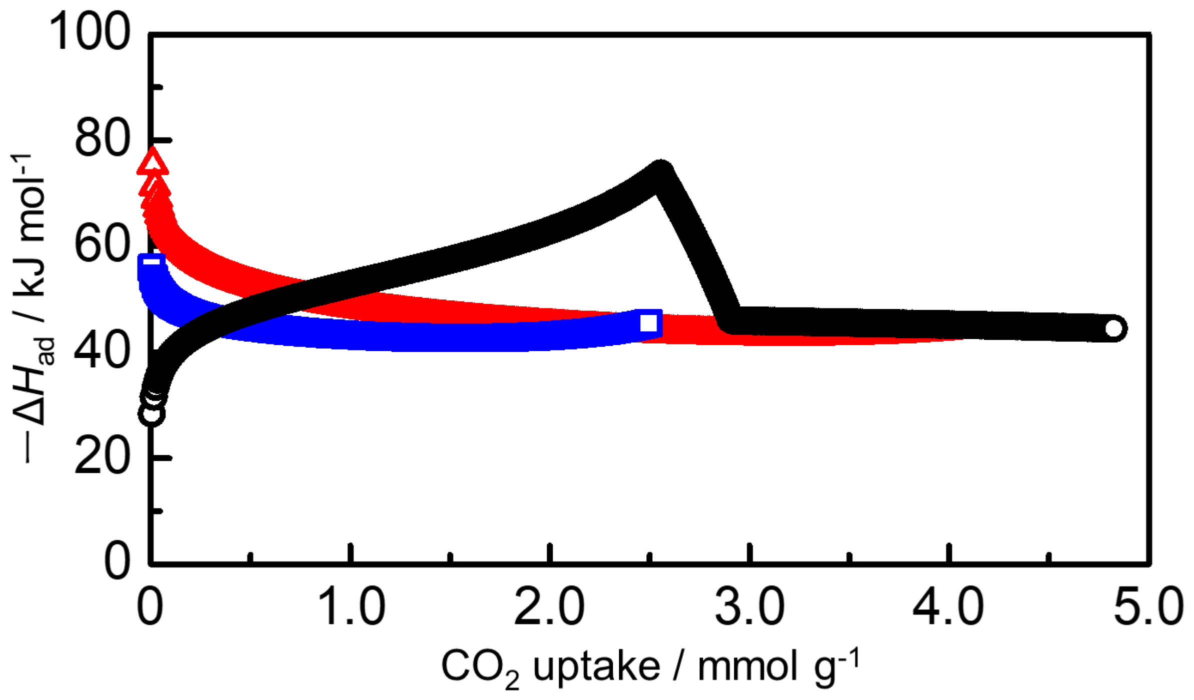

Figure S23. Isosteric adsorption enthalpy change of (○) Na^+-, (△) Li^+-, and (□) K^+-GME zeolites in the range from 298 K to 318 K determined using the CO_2 adsorption isotherms in Figure S22.

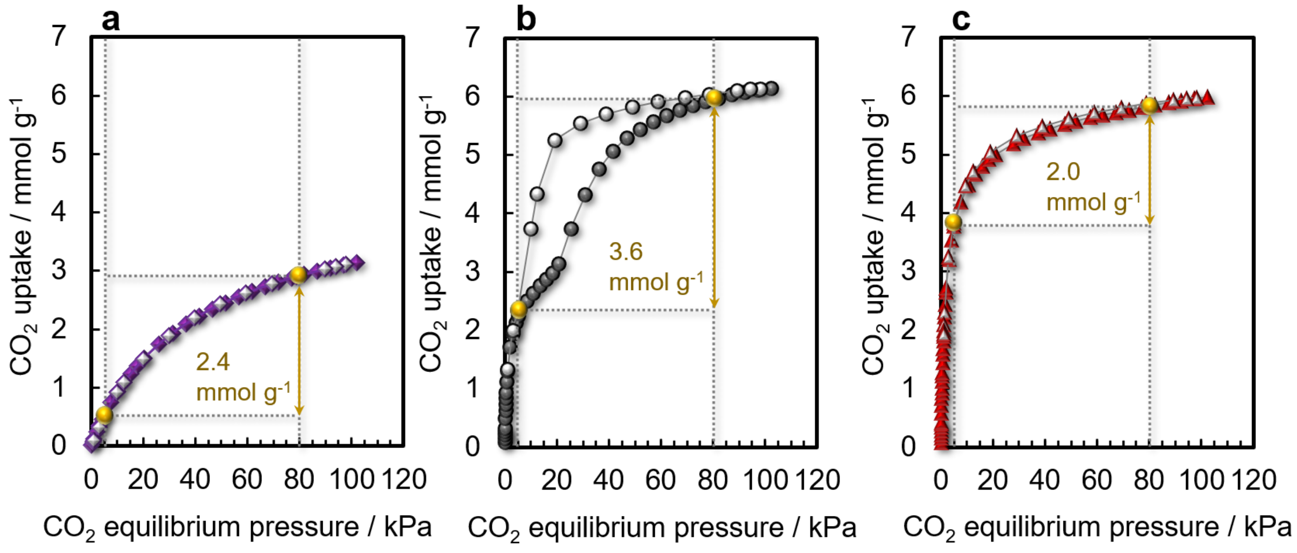

Figure S24. CO_2 working capacities of (a) Na^+-FAU, (b) Na^+-GME, and (c) Li^+-GME zeolites at 298 K in the pressure range of 5-80 kPa (closed symbol: adsorption, open symbol: desorption).
